# Capsid transfer of the retrotransposon Copia controls structural synaptic plasticity in *Drosophila*

**DOI:** 10.1371/journal.pbio.3002983

**Published:** 2025-02-18

**Authors:** P. Githure M’Angale, Adrienne Lemieux, Yumeng Liu, Shuhao Wang, Max Zinter, Gimena Alegre, Alfred Simkin, Vivian Budnik, Brian A. Kelch, Travis Thomson

**Affiliations:** 1 Department of Neurobiology, University of Massachusetts Chan Medical School, Worcester, Massachusetts, United States of America; 2 Department of Biochemistry and Molecular Biotechnology, University of Massachusetts Chan Medical School, Worcester, Massachusetts, United States of America; Stony Brook University Medical Center: Stony Brook University Hospital, UNITED STATES OF AMERICA

## Abstract

Transposons are parasitic genome elements that can also serve as raw material for the evolution of new cellular functions. However, how retrotransposons are selected and domesticated by host organisms to modulate synaptic plasticity remains largely unknown. Here, we show that the Ty1 retrotransposon *Copia* forms virus-like capsids in vivo and transfers between cells. *Copia* is enriched at the *Drosophila* neuromuscular junction (NMJ) and transported across synapses, and disrupting its expression promotes both synapse development and structural synaptic plasticity. We show that proper synaptic plasticity is maintained in *Drosophila* by the balance of *Copia* and the *Arc1* (activity-regulated cytoskeleton-associated protein) homolog. High-resolution cryogenic-electron microscopy imaging shows that the structure of the Copia capsid has a large capacity and pores like retroviruses but is distinct from domesticated capsids such as dArc1. Our results suggest a fully functional transposon mediates synaptic plasticity, possibly representing an early stage of domestication of a retrotransposon.

## Abstract 2

Transposons are parasitic genome elements that serve as raw material for the evolution of new cellular functions. In this study, the capsid of the virus-derived retrotransposon Copia is shown to mediate synaptic plasticity at the Drosophila neuromuscular junction.

## Introduction

Transposable elements (TEs) are DNA sequences that can move within the genome. TEs were once thought of as being “junk DNA,” and in ALS and Alzheimer’s *Drosophila* models suggest that TEs may be contributing to the pathologies of neurodegenerative diseases [1,2]. Despite these deleterious roles, there is considerable evidence that TEs provide genetic variability for the evolution of new molecular, cellular, and organismal functions. When a TE-derived DNA segment is selected for, its fitness benefits the host, and it can become a “domesticated” gene. Though it remains unclear how TE domestication occurs, particularly at the early stages.

Retrotransposons (RTEs) are robust sources of domesticated genes, especially in mammals [3]. They are selfish genetic elements that replicate using an RNA intermediate which becomes reverse transcribed into DNA for integration at another site. Thus, RTEs use a “copy and paste” strategy for replication to move and spread throughout the host genome. Approximately 40% of the human genome consists of RTEs, illustrating the effectiveness of their reproductive strategy though only a small subset of these elements remains active in the genome. The long terminal repeat retrotransposon replication mechanism is similar to that of retroviruses such as human immunodeficiency virus (HIV) and uses related proteins. For example, similar GAG proteins form capsid shells that protect and transport the RNA intermediate, and related enzymes such as reverse transcriptase and integrase are used to replicate the sequence and insert it into the genome. In fact, retrotransposons (RTEs) are endogenous retroviruses of the viral genus *Metaviridae* [4].

Recent work has revealed that the capsid regions of RTEs have been domesticated numerous times throughout Metazoan evolution. For example, we and others discovered that the gene Arc (activity regulated cytoskeleton-associated; dArc1 in *Drosophila*) is derived from a retroviral capsid region and retains the ability to form capsids [5,6]. These capsids carry RNA in extracellular vesicles across the neuromuscular junction (NMJ), which is important for synaptic plasticity and development. Thus, Arc has retained its viral-like behavior of RNA transfer to mediate its function in establishing synaptic plasticity. However, the dArc1 capsid structure is much smaller and less porous than retroviral counterparts [7], suggesting that dArc1’s mechanism has been specialized following its domestication.

The remarkable viral-like mechanism of Arc compelled us to examine how other capsid-forming elements may contribute to neuronal development and synaptic plasticity. We had previously found that RNA transcripts of *Copia*, an active retrotransposon, are also enriched in extracellular vesicles (EVs) at the NMJ [5]. Copia is known to form capsid-like structures [8–12], which prompted us to explore its relationship to neuronal development.

We found Copia can form capsids in a cell-free manner and that it associates with its own mRNA in vivo, suggesting that Copia retains its viral-like characteristics of trafficking its RNA. We developed a suite of tools, including antibodies and tissue-specific manipulation of *Copia* expression, and found that the capsid region of Copia is enriched at and transfers across *Drosophila* NMJs. Strikingly, disrupting *Copia* expression at the NMJ led to an increase in synapse formation and structural synaptic plasticity. We further found that Copia and dArc1 have an antagonistic relationship phenotypically and genetically. Because Copia behaves in a viral-like manner, we used cryogenic-electron microscopy (cryo-EM) to determine the high-resolution structure of the Copia capsid and found that it is distinct from the related dArc1 capsid, with a larger internal capacity and the presence of pores similar to those used by retroviruses to carry out their reverse transcriptase activity. The Copia capsid also contains a large amount of RNA, which we show is necessary for its assembly. Taken together, our observations suggest that Copia is a critical factor in NMJ plasticity that may interact with a well-established plasticity pathway. This novel form of TE domestication provides some of the first in vivo evidence that TEs play a pivotal role in neuronal development.

## Results

### Copia^gag^ is enriched in neurons and transfers across the *Drosophila* NMJ

**Fig 1:**
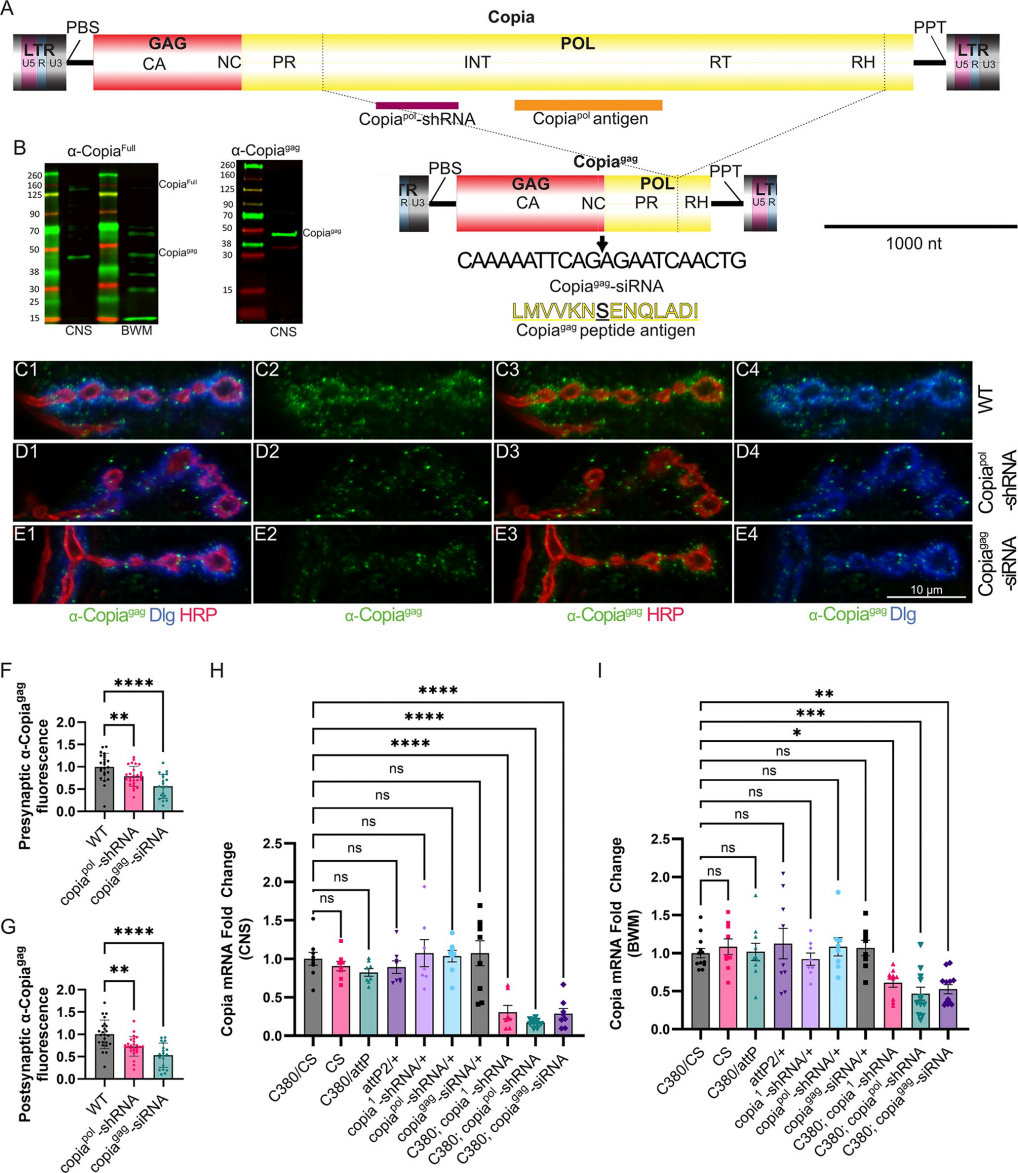
A spliced isoform of the retrotransposon *Copia* is enriched in neuronal tissue. (A) Representation of the entire *Copia* genome (*Copia*^full^) and the spliced form (*Copia*^gag^). The location of the RNAi targets, splice site (dotted lines), where the spliced ends are joined (thick black arrow) and the peptide used to generate the Copia^gag^ antibody are indicated. (B) Western blot of larval CNS and BWM lysate with the predicted size of Copia^gag^ indicated on the right and a molecular weight ladder on the left. (C) Representative confocal images of α-Copia^gag^ immunostaining show a striking enrichment at the wild-type *Drosophila* larval NMJ. (D, E) Knockdown with either *Copia*^pol^-shRNA or *Copia*^gag^-siRNA constructs expressed in the presynaptic compartment (C380-Gal4) cause a clear reduction of α-Copia^gag^ in both pre- and postsynaptic sides of the NMJ compared to wild type (C380-Gal4/Canton-S). (F) Quantification of presynaptic α-Copia^gag^ normalized immunofluorescence and postsynaptic (G) intensity of the same. Quantification of *copia* mRNA in the larval CNS (H) and BWM (I) by digital PCR of strains used in this study showing a significant decrease of *copia* mRNA in lines with both the Gal4 driver and *copia* si/shRNA. The data underlying the graphs shown in the figure can be found in S1 Data, raw gel images can be found in S1 Raw Images. DLG = α-Discs Large (postsynaptic marker), HRP = α-horseradish peroxidase (presynaptic marker). N = from left to right; number of animals/NMJs quantified, 14/27, 9/17, 9/16 in (F) and (G). WT is C380-Gal4/Canton-S for C panels. ns *p* ≥ 0.05, * *p* < 0.05, ** *p* < 0.01, *** *p* < 0.001, and **** *p* < 0.0001. Abbreviations: Gag: Capsid forming proteins, which contains capsid (CA) and nucleocapsid (NC) sequences. Pol consists of the following subdomains: Protease (PR), Integrase (INT), reverse transcriptase (RT), and RNase H (RH). ENV: Envelope. The Long terminal repeat region (LTR) is needed for transposable element replication, consisting of U5, R, and U3 sub-regions. PBS: Primer Binding Site. PPT: Polypurine tract.

To study the function of Copia in *Drosophila* EVs, we first developed an antibody against the full-length, unspliced Copia protein (α-Copia^Full^) that recognizes peptides encoded by the GAG and POL regions, and another antibody (α-Copia^gag^) against a peptide encoded by *Copia*^*gag*^, an alternatively spliced isoform of the *Copia* transcript (Figs 1A and S1A). Binding of α-Copia^gag^ to *Drosophila* Schneider 2 (S2) cell lysates was blocked by the presence of the antigen peptide, validating the antibody specificity (S1B Fig). We probed lysates of the larval central nervous system (CNS) and body wall muscles (BWMs) and observed that the predominant band corresponds in size to the predicted molecular weight of Copia^gag^ (Fig 1B). In contrast, in the BWM we see bands of varying sizes, which is consistent with auto-cleavage of the polyprotein. This is typically observed in retroviruses where the full-length polyprotein is cleaved into small functional peptides [13–16] and is consistent with the previously reported viral nature of TEs such as Copia [11]. These results indicate that the capsid-forming splice variant (Copia^gag^) is abundant and the predominant form of Copia in larval CNS tissue.

We next asked whether Copia capsid protein is transported across the NMJ. Immunolabeling with α-Copia^gag^ revealed a striking enrichment at the *Drosophila* NMJ (Fig 1C). To determine whether the Copia capsid protein is crossing the synapse, we leveraged the powerful Gal4/UAS system [17], which has been extensively used for precise genetic manipulation in pre- and/or postsynaptic cells at the NMJ. We designed 3 UAS-shRNA constructs targeting different regions of *Copia* (Fig 1A). We observed a substantial reduction of the α-Copia^gag^ signal when expressing *Copia*^*pol*^-shRNA specifically in motor neurons using the presynaptic C380-Gal4 expression driver (Fig 1D and 1F). Notably, we see reduced α-Copia^gag^ staining in the postsynaptic area abutting the synapses (Fig 1G). (See S1C Fig for a definition of the anatomy of a bouton, with pre- and postsynaptic areas highlighted.) This result strongly suggests that the postsynaptic localization of Copia^gag^ is at least in part due to a pool of Copia RNA that is derived from the presynaptic cell.

To determine whether Copia^gag^ is transferring across the synapse, we utilized a siRNA construct, UAS-Copia^gag^-siRNA, that recognizes the *Copia*^*gag*^ splice site (Fig 1A). The *Copia*^*gag*^-siRNA is predicted to specifically disrupt *Copia*^*gag*^ and not the full length unspliced isoform of *Copia*. We observed a substantial reduction of α-Copia^gag^ signal at the NMJ when expressing *Copia*^*gag*^-siRNA presynaptically (Fig 1C, 1E and 1G). We expressed both *Copia*^*gag*^-siRNA and the *Copia*^*pol*^-shRNA in the postsynaptic muscle using the C57-Gal4 expression driver and observed a decrease of α-Copia^gag^ signal in the postsynaptic area, but we did not see a decrease of α-Copia^gag^ signal presynaptically (S1D–S1H Fig). This result suggests that the transfer of Copia^gag^ is pre- to postsynaptic, but *Copia* mRNA can be affected by postsynaptic RNAi expression. Altogether this suggests that the postsynaptic localization of Copia^gag^ is at least in part due to a *Copia* mRNA pool derived from the presynaptic cell. We validated that this pattern was consistent at the mRNA level, as only larvae expressing a *Copia*-si/shRNA had a significant decrease in *Copia* mRNA expression (Fig 1H and 1I).

We sought to address whether our RNAi constructs exhibit off-target effects. TEs can co-splice with nearby genes, so it is possible that a TE-gene chimera could be repressed by one of our RNAi constructs [18]. We used whole genome sequencing to identify any TE-gene chimeras occurring in the *Drosophila* lines carrying our Gal4-UAS constructs. While we did identify rare chimeric sequences, our RNAseq data does not show any repression of the chimeric genes by our RNAi constructs (see **Methods**). Thus, we attribute the effects of our Gal4-UAS RNAi constructs specifically to *Copia* repression.

### Copia and dArc1 capsids are in mutually exclusive EVs

**Fig 2:**
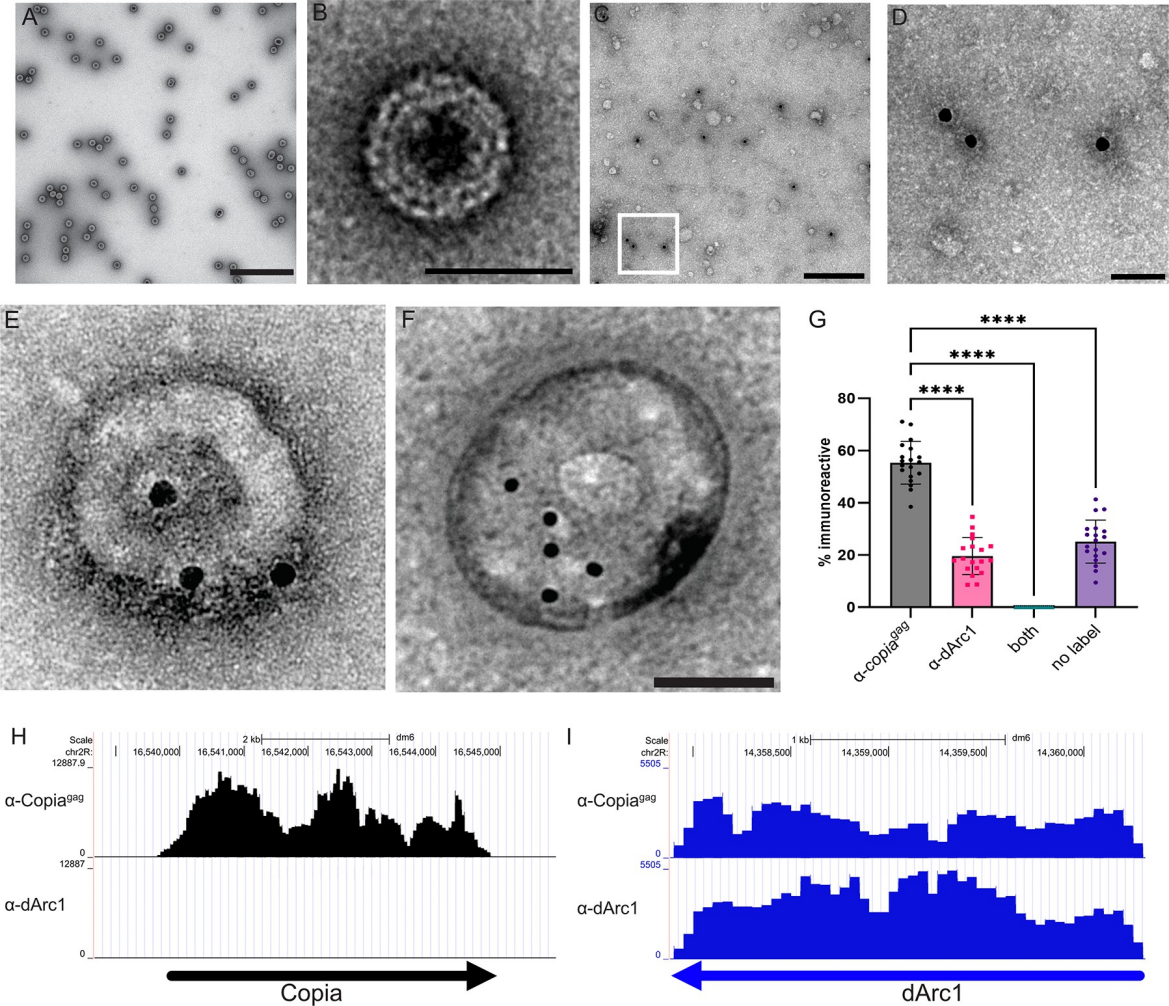
Copia is present in EVs, binds to *Copia* and *dArc1* transcripts, and is in mutually exclusive EVs as dArc1 capsids. (A) Bacterially expressed Copia^gag^ self-assembles into capsid-like structures, which are observable using negative stain EM, scale bar = 200 nm. (B) Close-up of an individual capsid, scale bar = 50 nm. (C) Representative EVs isolated from S2 cells were immuno-stained with α-Copia^gag^ (18 nm gold secondary). (D) A higher magnification of the closed square in D showing electron density of the α-Copia^gag^ stained S2 EVs (scale bar = 50 nm). (E, F) Representative EVs isolated from S2 cells were simultaneously immuno-stained with α-Copia^gag^ (18 nm gold secondary) (G), α-dArc1 (10nm), (H) scale bar = 50 nm. (G) Quantification of dual immuno-staining of 20 grids (representative images in 2C and 2D) were co-labelled with both α-Copia^gag^ and α-dArc1. Particles labelled with either α-Copia^gag^ or α-dArc1, both together, and unlabeled were counted (*n* = 1,042). (H, I) Mapping of RIP-seq, antibody used for immunoprecipitation on the y-axis, the genomic region of A is Copia and of B is dArc1. The data underlying the graphs shown in the figure can be found in S1 Data. EV, extracellular vesicle; RIP, RNA immunoprecipitation.

The spliced form of *Copia* (*Copia*^*gag*^) contains the GAG region that encodes the capsid protein and a small part of the POL region that consists primarily of the protease region necessary for auto-processing and viral maturation [14,15] (Fig 1A). Consistent with previous studies, we found that bacterially expressed Copia^gag^ auto-assembled into capsid-like structures [8–10,12,19] (Fig 2A and 2B). Copia^gag^ protein can form capsids of varying sizes, ranging from 30 nm to more than 120 nm in diameter (S1I and S1J Fig). The variable sizes of these capsids have important implications for capsid formation and entry into cells and/or nuclei.

We previously found that Copia^gag^ mRNA was enriched in EVs derived from *Drosophila* S2 cultured cells [5]. To determine whether Copia^gag^ capsids are also present in EVs, we isolated EVs from S2 cells, lysed them with detergent, and immunostained with α-Copia^gag^ then observed using EM. We observed electron densities consistent with capsids labelled with the Copia antibody, suggesting that these EVs contain Copia capsids (Fig 2C and 2D, quantified in 2G). Capsid proteins surround and protect the viral genome during its transfer between cells. Thus, we tested whether Copia^gag^ associates with *Copia* transcripts in vivo by conducting RNA immunoprecipitation (RIP) experiments using larval CNS and BWM extracts dissected from wild-type *Drosophila* third-instar larvae. We found that both α-Copia^Full^ and α-Copia^gag^ immunoprecipitated the *Copia* transcript from both of these compartments in vivo (S2A–S2F Fig). This data indicates that Copia forms capsids in vivo and encapsulates its own transcript.

We next investigated whether Copia and dArc1 capsids co-localize in EVs. We isolated EVs from S2 cells, exposed them to saponin, to permeabilize the EV membranes, but keep EVs intact. We then incubated these saponin-treated EVs with α-Copia^gag^ and α-dArc1 and imaged using immuno-EM. We found that there is a striking exclusion of Copia from dArc1-labeled EVs and of dArc1 from Copia-labelled EVs (Fig 2E and 2F**)**. We also double labeled some saponin treated EVs with Copiagag and Syntaxin-1A, a well-characterized EV marker in *Drosophila* and found that Copia and Syntaxin-1A co-labeled some of the EVs (S2G Fig). We further performed this experiment with harsher detergent treatments that remove the EV membranes (as in Fig 2C and 2D, quantified in Figs 2G, S2H, and S2J) and found that the capsid-like electron dense structures only label with either α-dArc1 or α-Copia^gag^. We performed secondary antibody controls and found no immunoreactivity (S2I Fig). We also found that electron densities that were labelled with Copia antibody were about 55 to 60 nm (S2J Fig) which is consistent with the size we observe as the predominant species of in vitro formed EVs (Figs 2B and S1J).

We next asked whether Copia or dArc1 interact with each other’s mRNA. We had previously observed that dArc1 protein does not precipitate *Copia* mRNA [5]. Consistent with this, sequencing of bound RNA following immunoprecipitation (RIP-Seq) using the α-dArc1 antibody did not show enrichment for *Copia* mRNA (Fig 2H). In contrast, Copia immunoprecipitated with both *Copia* mRNA and *dArc1* mRNA (Fig 2I). We confirmed the association of Copia^gag^ with both *Copia* and *dArc1* transcripts using dPCR (S2K–S2P Fig). Thus, our results indicate that dArc1 and Copia form separate capsid structures and traffic through separate EVs, but Copia capsids bind both *Copia* and *dArc1* mRNA while dArc1 only associates with its own transcript.

### Copia and dArc1 have an antagonistic relationship

**Fig 3:**
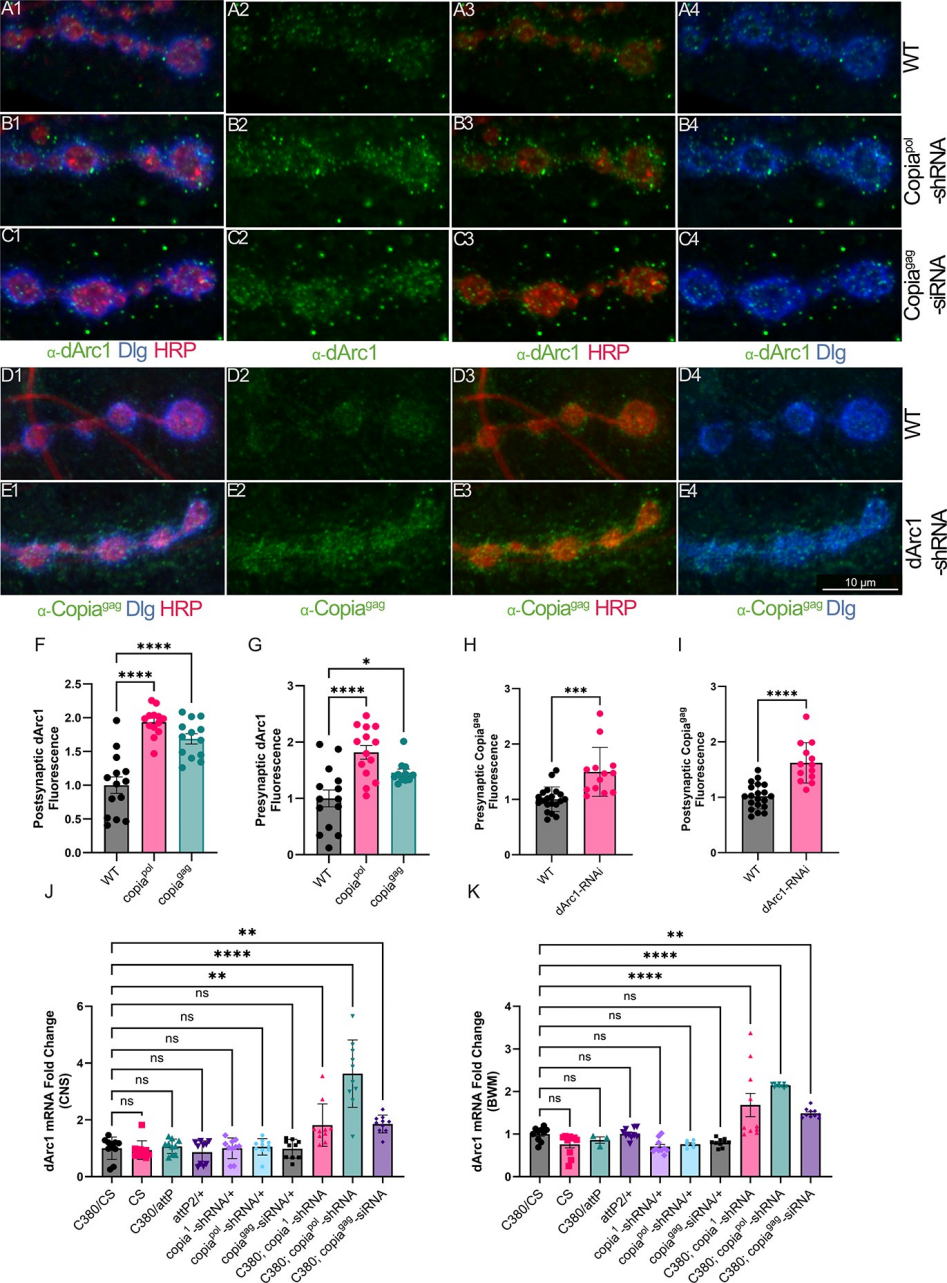
*Copia* and *dArc1* have an inverse relationship at the *Drosophila* NMJ. (A–C) The presynaptic knockdown of *Copia* with *Copia*^pol^-shRNA (B1–B4) and *Copia*^gag^-siRNA (C1–C4) leads to a significant increase in α-dArc1 staining both pre- and postsynaptically at the larval NMJ compared to wild type (A1–A4). (D, E) The presynaptic knockdown of *dArc1* (E1–E4) leads to a significant increase in α-Copia^gag^ staining both pre- and postsynaptically at the larval NMJ compared to wild type (D1–D4). (F–I) Quantification of the data represented in A–E. Quantification of *dArc1* mRNA in the larval CNS (J), and BWM (K) of strains used in this study, showing a significant decrease of *copia* mRNA in lines with both the Gal4 driver and *copia* si/shRNA. The data underlying the graphs shown in the figure can be found in S1 Data. DLG = α-Discs Large (postsynaptic marker), HRP = α-horseradish peroxidase (presynaptic marker). N = number of animals/NMJs quantified in (A) 8/14, (B) 8/14, (C) 8/14, (D) 11/20, and (E) 8/13. WT is C380-Gal4/Canton-S for panels A, D, and F–I. ns *p* ≥ 0.05, * *p* < 0.05, ** *p* < 0.01, *** *p* < 0.001, and **** *p* < 0.0001. BWM, body wall muscle; CNS, central nervous system; NMJ, neuromuscular junction.

Copia and dArc1 both form capsids that bind to their RNAs and are transferred across the NMJ through EVs. To test if the 2 proteins act antagonistically, we examined whether Copia levels affect dArc1 levels and vice versa. Presynaptic disruption of *Copia* using *Copia*^*pol*^-shRNA or *Copia*^*gag*^-siRNA led to a large accumulation of α-dArc1 signal both pre- and postsynaptically (Fig 3A–3C, 3F, and 3G). Additionally, presynaptic knockdown of *dArc1* in flies resulted in an increase of α-Copia^gag^ signal in the pre- and postsynaptic compartments (Fig 3D, 3E, 3H, and 3I). Thus, reduction of either *Copia* or *dArc1* results in increased protein levels of the opposing factor. Consistent with an increase of dArc1 protein at the NMJ in larvae expressing *Copia*-RNAi constructs, we observed an increase of *dArc1* mRNA in the pre- and postsynaptic tissue as determined by dPCR (Fig 3J and 3K). This suggests that the regulation of these opposing factors occurs at the mRNA level.

### The *Copia* capsid structure suggests the molecular basis for RNA encapsulation

Both dArc1 and Copia are retroviral-like factors that form capsids and have mutually antagonizing effects at the larval NMJ. To further investigate how Copia behaves in a virus-like manner, we sought to determine the three-dimensional structure of the *Copia* capsid. We expressed recombinant His6-SUMO-Copia^gag^ protein in *E*. *coli* and purified the protein to homogeneity (S3A–S3C Fig). We observed efficient auto-processing of the Copia^gag^ protein within the *E*. *coli* to form separate capsid (CA) and protease (PR) proteins, indicating that auto-processing does not require any *Drosophila-*encoded factors (S3A and S3B Fig). This result is similar to an earlier report showing auto-processing of full-length Copia polyprotein in *E*. *coli* [16]. Upon removal of the N-terminal His6-SUMO domain, we observed self-assembly of the Copia^gag^ protein into capsid-like particles of assorted sizes as well as some irregular aggregates.

**Fig 4:**
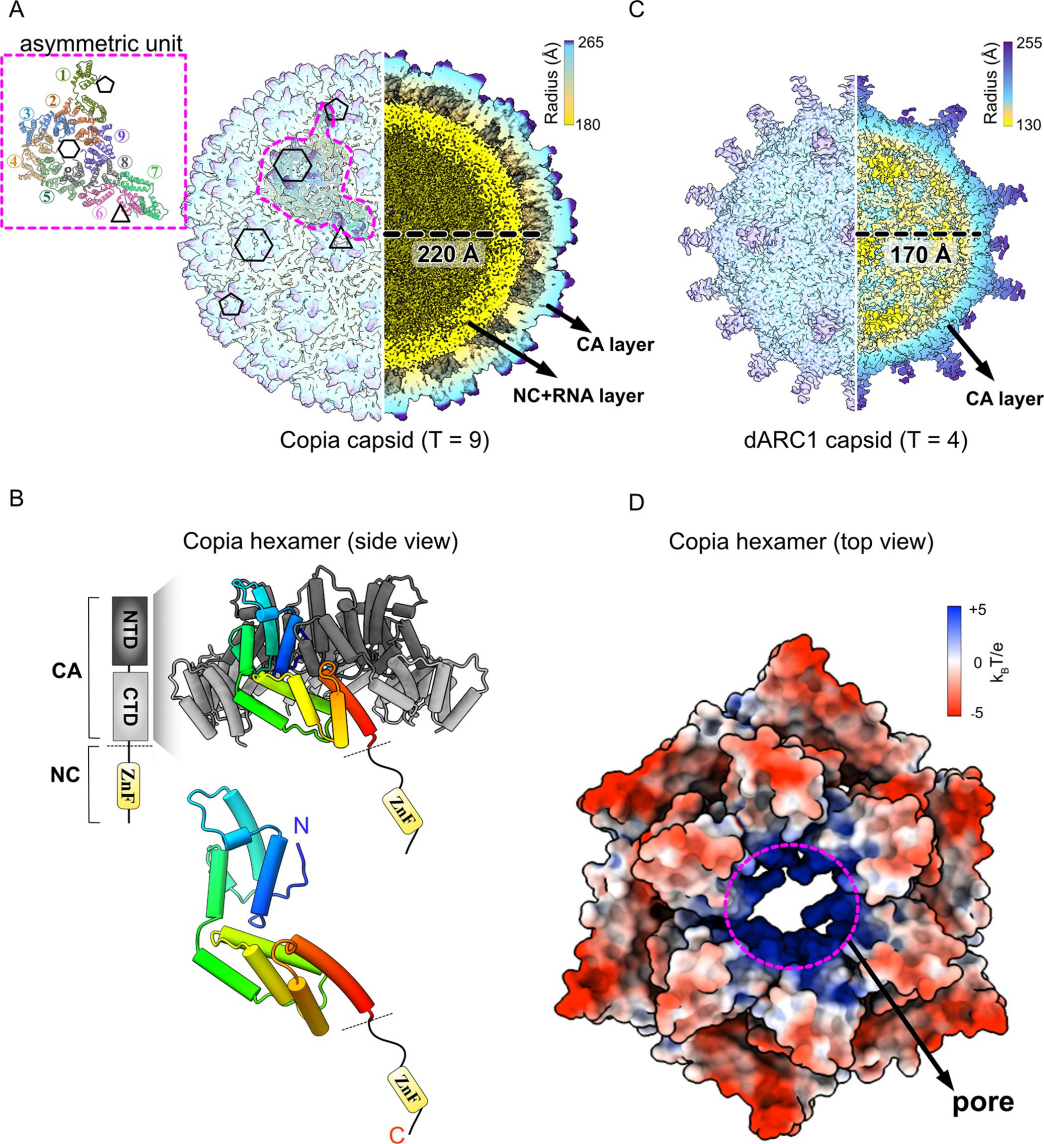
The cryo-EM structure of icosahedral Copia^Gag^ capsid. **(**A) The electron density map of the Copia^Gag^ capsid, colored by radius. The left half of the map is the surface of the capsid, and the right half of the map is a cross-section view. The 5-fold pentamer, 3-fold hexamer, and non-symmetric hexamer are denoted as black pentagon, triangle, and hexagons, respectively. The asymmetric unit is outlined in a magenta dashed line on the map and shown in detail on the top left panel, highlighting the 9 monomers composing one asymmetric unit (T = 9). The radius (220 Å) is measured between the center of the capsid and the mass center of the Copia^CA_CTD^ of the asymmetric unit. The outer layer is labeled as capsid (CA) layer, and the yellow inner layer density is labeled as nucleocapsid (NC) and RNA layer, the structure of which is undetermined due to disorder. (B) The atomic model of the Copia hexamer. Top panel: model of the CA region is colored with NTD in dark gray and CTD in light gray. The unstructured NC domain is shown as a black line with the zinc finger domain (ZnF) located near the C-terminus. One monomer within the hexamer is colored in rainbow from the N- to C- terminus. Bottom panel: the atomic model of one Copia^Gag^ monomer. (C) The electron density map of the dArc1 capsid colored by radius and was determined in a previous study [7]. The left half of the map is the surface of the capsid; the right half of the map is a cross-section view. The radius (170 Å) is measured between the center of the capsid and the mass center of the dArc1^CA_CTD^. The outer layer is labeled as capsid (CA) layer; the spikes on the surface of dArc1 capsid are unmodeled. There is no bound RNA in the assembled dArc1 capsid. (D) Copia^Gag^ non-symmetric hexamer is colored by electrostatic potential. The positively charged (blue) pore is at the center of the hexamer. The data underlying the structure shown in the figure can be found in S1 Data. CTD, C-terminal domain; NTD, N-terminal domain.

Our purification protocol enriched capsids of sufficient homogeneity such that we could determine their structure using single-particle cryo-EM methods (S4A Fig). Our reconstruction of the Copia^gag^ capsid when enforcing T = 9 icosahedral symmetry reached ~4.2-Å overall resolution (Figs 4A and S4B). There is a layer of featureless density lining the inner surface of the Copia capsid that likely represents the RNA that comigrates with the *Copia*-gag protein. Because the RNA does not adopt T = 9 symmetry, its density is amorphous in our reconstruction. To enhance the resolution of the capsid region, we used local reconstruction methods to determine the structures of individual subregions of capsid known as capsomers, which are sub-assemblies that are found at the vertices or faces of the icosahedron (S4D Fig). Local reconstruction of the pentameric capsomers found at the vertices provided a map to an overall resolution of ~3.3-Å. Because the *Copia* capsid adopts T = 9 geometry, there are 2 different hexameric capsomers along the faces (a hexamer with local 3-fold symmetry and a non-symmetric hexamer) and local refinement of each hexamer provided high-resolution maps (overall resolution of the non-symmetric and 3-fold symmetric hexamers are ~3.5-Å and 3.4-Å overall resolution, respectively). The high-quality maps of the locally refined capsomers allowed us to build an atomic model of the Copia^gag^ protein with high confidence and good stereochemistry (Figs 4B and S4C).

The capsid layer is composed of the Copia^gag^ protein’s CA region, which contains 2 alpha-helical domains (Fig 4B). The CA region is attached through a flexible linker to the nucleocapsid (NC) region, which harbors a Zn-finger RNA-binding domain. The NC region is not clearly visualized in the map but is located in the interior of the capsid and is associated with the density attributed to RNA. The N- and C-terminal domains (NTD and CTD) of the CA region are alpha-helical domains that are joined by a flexible linker. The NTDs form central spokes at the center of each capsomer sub-assembly, while the CTD is arranged in the capsomer periphery. The NTD and CTD of Copia are structurally similar to those seen in retroviruses and other retrovirus-like capsids, such as dArc1 (C_a_ RMSD ~3.5 Å and 2.7 Å for the NTD and CTD, respectively) [7].

The Copia capsid is quite different than that of Arc. The Copia capsid is much larger than that of the T = 4 dArc1 capsid (inner radii of ~220 Å and ~170 Å, respectively), resulting in ~2.2× increased internal capacity (Fig 4C). We hypothesize that the *Copia* capsid is larger than dArc1 because it packages larger cargo, such as the longer *Copia* mRNA (5 kbp versus 2 kbp) as well as the *Copia-*encoded enzymes reverse transcriptase and/or integrase. In addition to differences in size, Copia and dArc1 capsids are formed using quite distinct types of interactions, with very dissimilar electrostatic surface potentials (S5A Fig). This implies that Copia and dArc1 capsid proteins cannot physically interact with each other. Moreover, the Copia capsid also lacks the spike structures that protrude from the outer surface of the dArc1 capsid [7]. In place of the spikes, the *Copia* capsid contains positively charged pores at the center of each hexameric or pentameric sub-assembly, similar to HIV [20] (Fig 4D). The positively charged pores of HIV function as gates for dNTP entry into the capsid for fueling reverse transcriptase activity [21]. We hypothesize that the pores in the *Copia* capsid play a similar role. In support of this hypothesis, the capsids encoded by the domesticated GAG genes (*dArc1* and *PNMA2*) both display spikes that block these pores, presumably because dArc1 and PNMA2 capsids do not harbor an active reverse transcriptase [22]. In contrast, structures of active retrovirus or retrotransposon capsids exhibit open pores [20,23–25] presumably to facilitate reverse transcription.

Copia forms less interactions within a capsomer than other retroelement-derived capsids for which high-resolution structures are available (S5B Fig). For example, Copia capsomers have ~20% less interaction area than HIV capsomers [20]. The Copia interaction area is ~5% less than the modeled regions of the dArc1 capsomers, even though the dArc1 spike protrusions that constitute a massive inter-subunit interaction are unable to be modeled in the dArc1 capsid structure [7]. Despite the less extensive interfaces between capsid subunits in Copia, we observed an interesting interaction between adjacent CTDs that is not found in other retroelement-derived capsids (S5B Fig). We suspect this CTD-CTD interaction plays a key role in stabilizing Copia capsids by locking down the periphery of each capsomer sub-assembly. The Ty3/Gypsy capsid also has this CTD-CTD interaction [25], suggesting that it is shared among the Ty1 and Ty3 families of retrotransposons (S5B Fig).

Because the Copia capsid structure exhibits less extensive interactions than other related capsids, we sought to determine the requirements for Copia capsid assembly. We found that *Copia* capsid formation requires the presence of RNA, as removing bound RNA using anion-exchange chromatography inhibited capsid assembly (S3C and S3D Fig). Likewise, deleting the RNA-binding Zn-finger domain (i.e., the *Copia*^gag^-ΔNC variant) inhibited capsid assembly (S3E Fig). However, the *Copia*^gag^-ΔNC variant was capable of co-assembly into capsid-like particles in the presence of WT-*Copia*^gag^ carrying RNA (S3F Fig). Thus, the *Copia*^gag^-ΔNC variant is assembly-competent as long as RNA-binding is achieved by some of the co-assembling subunits. These results establish a requirement for RNA in normal Copia capsid assembly. This is distinct from dArc1 capsids that can assemble in the absence of RNA [7], or mammalian Arc which assembles in absence of RNA albeit much less efficiently than with RNA bound [6]. Copia’s RNA requirement is much more similar to HIV, which requires RNA binding for assembly [26]. These results further demonstrate that *Copia* is functioning in a virus-like/retrotransposon fashion.

### Copia is a negative regulator of acute structural synaptic plasticity

**Fig 5:**
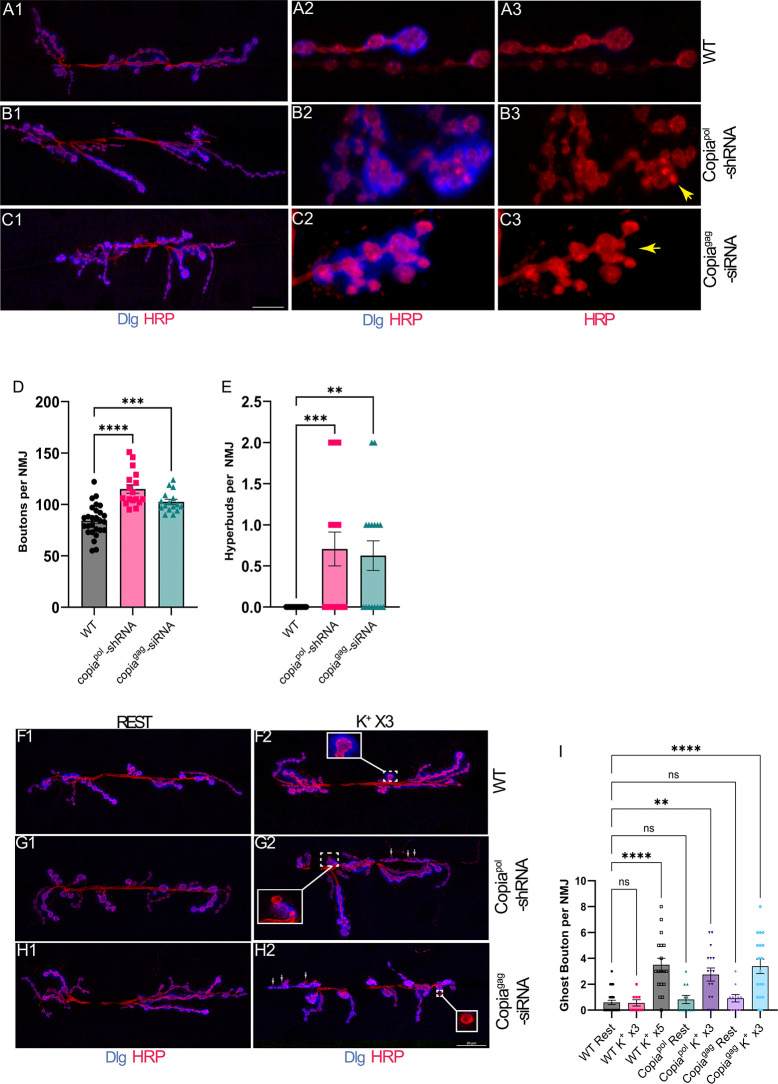
Disruption of *Copia* in motor neurons causes changes in synaptic development and plasticity. (A–C) Expressing *Copia*^pol^-shRNA (B) or *Copia*^gag^-siRNA (C) constructs in the motoneurons of larvae causes increased bouton formation at NMJs compared to wild type (C380-Gal4/Canton-S) (A). There is also a substantial increase in hyperbudding (A3 vs. B3 and C3), where 2 or more boutons “bud” off the same central bouton (yellow arrows designate budding boutons in B3 and C3). (D) Quantification of bouton numbers. (E) Quantification of hyperbudding events. (F–H) Stimulating wild-type flies (F) with 3 rounds of potassium treatments does not induce them to form new synapses (ghost boutons) but does induce increased bouton formation after disrupting expression of Copia (G and H). (I) Quantification of potassium stimulation in different genetic backgrounds. The data underlying the graphs shown in the figure can be found in S1 Data. DLG = α-Discs Large (postsynaptic marker), HRP = α-horseradish peroxidase (presynaptic marker). N for D and E = from left to right; number of animals/NMJs quantified, 14/27, 9/17, 9/16, for I N = 12/19, 10/13, 6/12, 9/17, 6/11, 9/18. WT is C380-Gal4/Canton-S for panels A and F. ns *p* ≥ 0.05, * *p* < 0.05, ** *p* < 0.01, *** *p* < 0.001, and **** *p* < 0.0001. NMJ, neuromuscular junction.

Because Copia^gag^ is enriched at the NMJ, we investigated its potential role in synaptic development and plasticity. Synaptic boutons at the NMJ are continuously formed throughout larval development [27]. Thus, an increase or decrease in the levels of bouton formation is an indication of developmental synaptic plasticity [28]. When we express *Copia*^*pol*^-shRNA with the neuronal C380-Gal4 driver to specifically reduce *Copia* expression presynaptically, we observe a striking ~50% increase in synaptic bouton number compared to the C380-Gal4 driver alone control (Fig 5A–5D). This effect was also seen with the single siRNA construct directed against the *Copia*^*gag*^ mRNA splice site (Fig 5A, 5C, and 5D). In addition to an enhancement in the number of synaptic boutons upon presynaptic expression of either *Copia*^*pol*^-shRNA or *Copia*^*gag*^-siRNA, we also observed an increase in “hyperbudding,” which we defined as the presence of 3 or more boutons budding off from a central, larger (parent) bouton (Fig 5E). Thus, *Copia* negatively regulates developmental plasticity, while *Arc* is a positive regulator. Consistent with this, overexpression of *dArc1* at the NMJ increased the number of boutons, which shows that elevated levels of dArc1 can overcome Copia-induced inhibition of bouton formation (S6F Fig).

As dArc1 and Copia have an inverse relationship such that reduction of one at the NMJ leads to an increase of the other, we assessed if *dArc1* and *Copia* genetically interact to determine their functional relationship. We disrupted *Copia* expression using either *Copia*^*pol*^-shRNA or *Copia*^*gag*^-siRNA in a *dArc1*-null mutant background. There was a substantial decrease in bouton formation in *dArc1*-null mutants, which was consistent with our previous studies [5] (S6F Fig). Reduction of *Copia* in neurons using either *Copia*^*pol*^-shRNA or *Copia*^*gag*^-siRNA in the *dArc1*-null background resulted in a substantial increase in bouton numbers and hyperbudding (S6A–S6G Fig), similar to levels observed with Copia^pol^-shRNA or Copia^gag^-siRNA expression in a wild-type background. These results are consistent with *Copia* having a predominant role at the NMJ over *dArc1* (S6H–S6K Fig).

### Copia is a negative regulator of acute structural synaptic plasticity

Our discovery that Copia is a negative regulator of bouton formation, which is an indication of developmental plasticity, prompted us to test if Copia has acute roles in regulating plasticity. New synaptic bouton formation can be stimulated in dissected larval NMJs with acutely repetitive spaced cycles of nerve stimulation with potassium (High K^+^; 90 mM) [29]. These nascent boutons, however, do not properly develop pre- and postsynaptic structures, and are thus called ghost boutons. In previous work, we found that NMJs from *dArc1*-null flies were less responsive to spaced K^+^ stimulation, which resulted in decreased formation of ghost boutons compared to controls [5]. Since larvae expressing Copia^pol^-shRNA and Copia^gag^-siRNA already have a larger number of synaptic boutons, Copia knockdowns have increased structural synaptic plasticity.

The number of cycles of spaced K^+^ stimulation required for ghost bouton formation can be used as a measure of acute structural synaptic plasticity [29]. Therefore, we sought to assess whether *Copia* knockdown altered the threshold for K^+^ stimulated formation of ghost boutons. As expected for wild-type *Drosophila*, 3 cycles of spaced K^+^ stimulation are insufficient to initiate ghost bouton formation, while 5 cycles induce robust ghost bouton formation (Fig 5F–5I). However, disrupting *Copia* expression presynaptically induced significant ghost bouton formation with only 3 cycles of K^+^ stimulation (Fig 5F–5I). This result shows that presynaptic reduction of *Copia* increases acute plasticity.

## Discussion

In this work, we present the first evidence that an active TE, Copia [30], is a potent regulator of structural synaptic plasticity. Interestingly, Copia and dArc1 seem to have an antagonistic relationship, providing evidence that the 2 capsid-encoding genes interact genetically to mediate plasticity at the fly NMJ. Copia is an inhibitor of synapse development, while dArc1 stimulates synapse formation. Proper synapse development therefore is a balance of these 2 opposing forces.

The details of this mechanism require further investigation. *Copia* controls plasticity, but is it dependent on the transfer of Copia capsids? We determined that EVs from S2 cells contain a detergent resistant electron dense structure that immunoreact with Copia antibodies, and further we found EVs immunoreacted with Copia antibodies, which strongly suggests that in S2 cells Copia is loaded into EVs presumably as a capsid. We also determined that presynaptic knockdown of Copia affects postsynaptic levels of Copia suggesting that Copia transfers across the synapse. However, work continues to determine if Copia, dArc1 or mammalian homologs of the latter transfer across synapses in a capsid in neurons. With the tools we have previously developed, and in this work, we will test if there are capsid-like structures at a synapse and whether they immunoreact with dArc1 and/or Copia antibodies, to confirm that the transfer of capsid-like structures is occurring at synapses.

The Copia knockdown phenotype is predominant to dArc1 for synaptic development at the larval NMJ, suggesting that dArc1 acts as an inhibitor of Copia, which is itself an inhibitor of plasticity. However, this does not reveal how this interaction might be carried out at the molecular level. A possible molecular mechanism is that each protein prevents assembly of the opposing capsid by binding to an assembly intermediate and blocking further oligomerization. We call this the “Poison Pill” hypothesis. We suggest this because both Copia and dArc1 form capsids using a related protein fold, which might indicate similar mechanisms. However, a challenge to this hypothesis is the distinct interaction surfaces within the Copia and dArc1 capsids (S5A Fig). Moreover, Copia and dArc1 do not co-immunoprecipitate, which indicates that they do not bind to each other or a shared intermediate simultaneously.

Another possible mechanism is that these capsids antagonize each other at the level of cargo transport. We believe that the currently available data is consistent with this model. For example, we show that Copia capsids require RNA for assembly, likely because Copia capsid proteins are weakly associated and the RNA acts as a “glue” to keep the capsid together. This would make Copia capsids exceptionally sensitive to the amount of RNA that is available. Furthermore, we observed that dArc1 and Copia have different RNA-binding capacities as determined by RIP-seq. Specifically, we see that dArc1 binds its own transcript but not *Copia* mRNA, while Copia binds its own transcript as well as *dArc1* mRNA. Copia binding *dArc1* mRNA this may sequester the dArc1 transcript from dArc1 capsids, thus limiting dArc1 function (S6I–S6K Fig). However, if dArc1 binds to its transcript with a higher affinity than Copia does, it would thereby protect dArc1 *mRNA* from Copia sequestration while reducing the available RNA substrate necessary for Copia capsid assembly (S6K Fig). Future experiments will investigate these possibilities.

Regardless of the nature by which Copia and dArc1 antagonize each other, the Copia structure suggests that it is substantially weaker than other retroviral or similar capsids. We observe that Copia capsids assemble through less extensive protein–proteins interactions than observed in most other retroelement-derived capsids. In general, capsid stability is controlled by the totality of interactions and the topological arrangement of the capsid subunits [31,32]. Because the topology of protein–protein interactions is identical across all retroelement-derived capsids, the total interaction area is likely to be a good proxy for overall capsid stability. Thus, we hypothesize that Copia capsids are less stable than most other retroelement derived capsids, thus requiring RNA to assemble. We note that the Ty3/Gypsy retrotransposon capsid has been documented to be relatively weak [25], indicating that weaker capsids might be a common feature of retrotransposons. It seems likely that the weaker capsids are related to the diverse morphologies observed in preparations of Copia and Gypsy capsids. It is possible that the weaker capsids are selected as part of the nuclear import mechanism. Klumpe and colleagues shows that the nuclear pore complex (NPC) selects for certain sizes of capsid to enter, thus we speculate that this weaker association may allow Copia capsids to re-arrange to better fit through nuclear pores [33].

Additionally, it is not clear how Copia is affecting plasticity and bouton formation. We observe postsynaptic Copia is dependent on presynaptic expression, also we observe that Copia forms capsids, and in S2 cells load these capsids into EVs. This suggests Copia forms a capsid, crosses the synapse, likely in an EV. Altogether, this leads to the exciting possibility that Copia may control the postsynaptic muscle in a non-cell autonomous manner by affecting processes in the nucleus such as transcription. A long-speculated way this could occur is by insertion of a TE into the genome. This could change the nearby chromatin structure or introduce novel transcriptional elements (i.e., promoters or repressors), thereby affecting gene transcription, which would be consistent with the somatic mosaicism described by Barbara McClintock [34]. This would require the full Copia machinery, including the reverse transcriptase and the integrase proteins. In support of this hypothesis, the Copia capsid structure shows pores like those of retroviral capsids that are used for transporting the dNTPs that are necessary for reverse transcription [20]. Our hypothesis is also supported by Klumpe and colleagues that resolves T = 9 Copia capsids in the nucleus [33]. It remains to be seen whether Copia inserts into the muscle genome, if these insertions are variant or not, and if this affects nearby gene expression. Alternatively, it may be possible for viruses or TEs [35] to affect gene expression without inserting into the genome through formation of nuclear aggregates. Regardless, the role of a TE in synaptic development needs to be further investigated to better understand synaptic plasticity as well as explore the potential functions of retrotransposons in directing host biology.

It is unlikely that Copia and dArc1 are the only capsid-encoding proteins that are transferred between cells, so there may be additional proteins that are functioning in a similar mechanism in the nervous system or other tissues. The *Drosophila* genome, like other eukaryotic genomes, has a huge repository of RTEs that could be used to modulate physiological functions with a capsid delivery-like mechanism similar to dArc1 and Copia. Recently, the mammalian capsid-like protein PEG10 was engineered to transfer RNA cargo, providing more evidence that the viral synaptic transfer of RNA (ViSyToR) pathway described in Ashley and colleagues is relevant beyond the *Drosophila* NMJ [36]. While the engineered capsid is direct evidence that PEG10 plays a role transporting RNA transcripts between cells, it suggests that a capsid-encoded gene can act in a viral-like manner. Together, these studies along with our discovery that the TE Copia regulates synaptic plasticity raises the likely possibility that dArc1 is one of a family of capsid-encoding genes that regulate cellular functions in neurons and throughout the body.

There are many examples of TE domestication, including dArc1, whereby a TE fragment is selected through evolution for its beneficial role for host fitness. However, until recently there have been few examples of an entire transposon is domesticated. Specifically, examples of this are the Cer1 gene of *C*. *elegans* and the *Line-1* elements that have a role in early mouse embryonic development [37,38]. While it has long been speculated that TEs have a role in neuronal development, and there is data that expression of *Line-1* in mammalian brains is developmentally regulated, no specific roles for *Line-1* or any other TEs in mammalian neurons have been identified [39–41]. As such, the recent discovery of a TE regulating neuronal cell migration in zebrafish [42] and our discovery of a TE regulating synaptic formation and plasticity suggest that we are at the beginning of an exciting new era of research uncovering the physiological roles of enigmatic sequences such as TEs. While most previous studies of TE domestication have focused on late stages of domestication (such as with *dArc1*), our discovery of Copia’s role in synaptic plasticity opens the door to study domestication at earlier stages. What factors determine whether a full-length TE or a subset of a TE is domesticated? What does it mean to be domesticated? We envision that these and other exciting questions will begin to be addressed by the study of Copia.

## Methods

### Experimental model and subject details

The following fly lines were used in this study: UAS-Copia^pol^-shRNA (see below), UAS-Copia^Gag^-siRNA (see below), UAS-dArc1-RNAi2 [5], w; dArc1^esm18^ (RRID:BDSC_37530, Bloomington *Drosophila* stock center, BDSC), y[1] w[67c23]; P(y[+t7.7] = CaryP0attP2) (RRID:BDSC_8622, Bloomington *Drosophila* stock center, BDSC), Canton-S (1, BDSC), C380-Gal4 [43] and C57-Gal4 [43]. Female third-instar larvae were used for all NMJ dissections.

### Fly husbandry

All flies were raised on low yeast molasses formulation *Drosophila* food at either 25°C or 29°C (Gal4/RNAi crosses).

### Constructs

For *Copia*^*pol*^-shRNA and other dsRNA constructs, the insert was synthesized (see S1 Data for the construct sequence), then cloned into pwallium10-roe as described in Ni and colleagues [44]. For the *Copia*^*gag*^ siRNA, forward and reverse primers were synthesized (see S1 Data for the construct sequence), annealed and cloned into pwallium20 as described in Ni and colleagues [44]. All constructs were injected into flies and site-directed insertion was accomplished using phiC-31 integrase, a service performed by Bestgene.

### Immunocytochemistry and antibodies

*Drosophila melanogaster* third instar larva body wall muscles were dissected in calcium-free saline and fixed in either Bouin’s fixative (0.9% (v/v) picric acid, 5% (v/v) glacial acetic acid, 9% (w/v) formaldehyde) or 4% (w/v) paraformaldehyde in 0.1 M phosphate buffer, pH 7.2. Fixed samples were washed and permeabilized in PBT (0.1 M phosphate buffer; 0.2% (v/v) Triton X-100) and incubated in a primary antibody overnight at 4°C. The samples were then washed 3 times with 1× phosphate-buffered saline (PBS) with 0.05% Tween-20 (PBT), incubated with secondary antibodies for 2 h at room temperature, washed 3 times, and then mounted in Vectashield Hardset Mounting Media (Vector Laboratories Inc.). The following antibodies were used: rabbit anti-Copia^Full^, 1:1,000 (see below), rabbit anti-Copia^Gag^, 1:5,000 (see below), rabbit anti-dArc1, 1:500 [5], rabbit anti-DLG, 1:40,000 [45], and mouse anti-DLG, 1:200. DyLight-conjugated and Alexa Fluor-conjugated secondary antibodies were obtained from Jackson ImmunoResearch (DyLight-405-conjugated goat anti-HRP, Alexa Fluor-594-congugated goat anti-HRP, Alexa Fluor-488-congugated donkey anti-Rabbit, Alexa Fluor-594-congugated goat anti-rabbit, Alexa Fluor-647-congugated goat anti-Mouse) and were used at 1:200, as described above.

Copia^Full^ antibodies were generated against a Copia antigen (see Fig 1) by immunizing rabbits with then entire Copia^gag^ protein (Pocono Rabbit Farm and Laboratory), while the Copia^gag^ antibodies were generated against a Copia peptide antigen (see Fig 1) by immunizing rabbits with the peptide LMVVKNSENQLADIC (GenScript).

### Activity paradigm

Potassium stimulations were carried out as described in Ataman and colleagues [29]. Larva were dissected in low-calcium (0.1 mM) HL3 saline [46], then pulsed with a series of high potassium (90 mM) saline; each pulse was spaced out by a 15-min rest period in low-calcium HL3 saline. The 5-cycle potassium stimulation consisted of three 2-min pulses, one 4-min pulse, and one 6-min pulse; followed by a final 15-min rest. The 3-cycle, subthreshold, potassium stimulation consisted of three 2-min pulses followed by a 15-min rest period. Following the 90-min pulse-rest cycle, samples were fixed with 4% paraformaldehyde and processed for immunocytochemistry as described above.

### Confocal microscopy and signal intensity measurements

Z-stacked images were acquired using a Zeiss LSM 800 confocal microscope equipped with a Zeiss 63X Plan-Apochromat 1.40 NA DIC M27 oil immersion objective and a Zeiss 40X Plan-Apochromat 1.30 NA DIC (UV) VIS-IR M27 oil immersion objective. After image acquisition with identical settings, the images were quantified as previously described [47]. In brief, volumetric measurements of the boutons of interest bound by HRP staining were selected and fluorescence intensity inside was measured using Volocity software (Quorum Technologies Inc.). The postsynaptic area was calculated by dilating the presynaptic area by 8 iterations and comparing to DLG staining and the HRP containing volume was subtracted and the intensity within the remaining volume was measured. Intensity was normalized to HRP bouton volume and data normalized to wild-type values.

We found that the knockdown of *dArc1* caused the α-Copia^gag^ staining to be saturated. To collect data at a linear intensity, we normalized the saturation points to the *dArc1*-siRNA samples rather than not wild type. Thus, the control samples appear to have a lighter intensity for this experiment.

### RNA immunoprecipitation

Wild-type *Drosophila* third-instar larvae were dissected, and the CNS and BWM were collected in separate tubes containing RIPA buffer (Abcam) supplemented with protease inhibitors (Roche) and RNase inhibitor (Invitrogen) in a manner previously described [5]. Similarly, S2 cells were grown to confluency, washed with ice-cold Dulbecco’s PBS (DPBS; Sigma), and resuspended in RIPA buffer. Tissue and cell lysates were homogenized using 0.5-mm glass beads at 4°C using a Bullet Blender 24 Gold homogenizer (Next Advance Inc.). Lysates were then centrifuged at 4°C to remove cell debris. Supernatants were precleared against Protein A/G magnetic beads (Pierce) and then incubated overnight at 4°C with either anti-Copia^Full^, anti-Copia^gag^, anti-dArc1 antibodies, or equal amounts of pre-immune serum. Samples were then incubated for 2 h at 4°C with protein A/G magnetic beads and washed several times with RIPA buffer. For immunoblotting, beads were incubated directly with 4× protein loading buffer (Li-Cor) with 2-Mercaptoethanol (Sigma). For digital PCR, RNA was eluted from the beads with RLT buffer (QIAGEN) supplemented with 2-mercaptoethanol and then purified using the RNeasy mini kit (QIAGEN) for the QIAcube connect (QIAGEN) with DNase digest using RNase-free DNase set (QIAGEN).

### Digital PCR (dPCR)

RNA samples were reverse transcribed into cDNA using the Superscript IV first-strand synthesis reaction (Invitrogen) following manufacturer protocol with RNase H digest. The dPCRs were multiplexed in 26K 24-well or 8.5K 96-well QIAcuity nanoplates (QIAGEN) using a QIAcuity system (QIAGEN). For the reactions, either QIAcuity evagreen master mix or probe master mix (QIAGEN) were used with the gene-specific primer sets for dArc1, Copia^Full^, Copia^gag^, Rpl32, and/or 18S rRNA or their probes (Thermo Fisher or IDT) (see S1 Data for sequences). Data was processed in the QIAcuity Software Suite (QIAGEN) where absolute values (copies/μl) were obtained and normalized expression derived.

### Immunoprecipitation

Third-instar wild-type *Drosophila* larvae were dissected in ice-cold Ca2+-free saline, and CNS and BWMs were homogenized as above. For S2 cells, they were grown to confluency, washed in ice-cold DPBS (Sigma), resuspended in RIPA buffer (Abcam) supplemented in protease inhibitor cocktails (Roche), and homogenized as above. Lysates were centrifuged at maximum speed at 4°C for 10 min. Protein concentration was determined by Qubit protein assay (Invitrogen) in a Qubit 4 fluorometer (Invitrogen). Supernatants were incubated with the respective antibody overnight at 4°C with gentle rotation, the protein-antibody complexes were incubated with protein A/G magnetic beads (Pierce) for an hour at room temperature. They were washed with buffer several times with final wash being in pure water. The magnetic beads were eluted with protein sample buffer at room temperature for 10 min with gentle rotation or boiled at 95°C for 10 min.

### Western blotting

Immune complexes from RIP and IP experiments were incubated at room temperature or 95°C for 10 min, proteins were separated in Mini-Protean TGX stain-free 4% to 20% precast gels (Bio-Rad) under reducing and denaturing conditions. Proteins were transferred to an Immun-Blot LF PVDF membrane (Bio-Rad) on a semi-dry Trans-Blot Turbo transfer system (Bio-Rad), blocked in Intercept blocking buffer (Li-Cor) and incubated with primary antibodies diluted in Intercept antibody diluent (Li-Cor) overnight at 4°C. Blots were washed, incubated with IRDye secondary antibodies (Li-Cor), washed again, and finally imaged on a Li-Cor odyssey CLx imaging system.

### Expression, purification, and assembly of Copia^Gag^ capsids

The short form of Copia encodes Copia^Gag^ and the protease domain (PR), which is termed Copia^Gag+PR^ in this study. To test if Copia^Gag+PR^ can form capsid-like particle through Copia^Gag^ self-assembly, we expressed and purified the Copia^Gag+PR^ protein using the *E*. *coli* expression system. Copia^Gag+PR^ was fused to an N-terminal His_6_-SUMO tag and cloned into the pSMT3 vector. pSMT3_His_6_-SUMO- Copia^Gag+PR^ was transformed into *E*. *coli* BLR (DE3) cells for protein expression. *E*. *coli* cells harboring pMST3_His_6_-SUMO- Copia^Gag+PR^ were cultured in 1 L Terrific Broth (containing 30 mg/L Kanamycin) at 37°C with shaking. When cells reached an optical density (600 nm) of 0.6 to 0.8, the culture was induced by adding isopropyl β-D-1-thiogalactopyranoside (IPTG) to the final concentration of 0.2 mM. The induction was done at 18°C with shaking for 24 h. After induction, cells were harvested by centrifuging at 5,000×g for 20 min at 4°C.

During induction, the protease domain in Copia^Gag+PR^ auto-processes the His_6_-SUMO- Copia^Gag+PR^ polyprotein to generate His_6_-SUMO- Copia^Gag^ which was purified using Ni-affinity chromatography. All the following purification steps were performed at 4°C. The cell pellet was resuspended in buffer A (25 mM HEPES, pH 7.4, 150 mM NaCl, 10% Glycerol, 100 μm ZnCl_2_, 2 mM 2-mercaptoethanol) supplemented with 20 mM imidazole and protease inhibitors. Cells were lysed by high pressure cell disruption on ice. Cell lysate was clarified by centrifuging at 20,000×g for 40 min at 4°C. The supernatant was transferred and filtered through a 0.45 μm PVDF membrane before loading onto the Ni-affinity column (Cytiva, HisTrap HP). His_6_-SUMO- Copia^Gag^ was eluted off the Ni-affinity column through a gradient of 20 to 500 mM imidazole. Only elution fractions with imidazole concentration from 400 to 500 mM were pooled and digested with His_6_-tagged SUMO-protease Ulp1 to cleave off the N-terminal His_6_-SUMO tag and trigger Copia^Gag^ capsid assembly. Copia^Gag^ capsids were further purified by removing free His_6_-SUMO and Ulp1-His_6_ by passing the protein across the Ni-affinity column. The His_6_-SUMO-Copia^GagΔNC^ construct was generated by mutating residue H186 to a stop codon in the wild-type pSMT3_His_6_-SUMO- Copia^Gag+PR^ plasmid using Quikchange mutagenesis. His_6_-SUMO-Copia^GagΔNC^ was induced and Copia^GagΔNC^ was purified the same way as the wild-type Copia^Gag^ described above.

### Copia^Gag^ protein RNA depletion

To remove the RNA from the purified Copia^Gag^ protein, Ni-affinity purification was performed as described above. After purification, the pooled protein fractions were immediately loaded onto an anion exchange column (Mono Q 5/50 GL, Cytiva), and protein was eluted off the column through a gradient of 150 to 1,000 mM NaCl supplemented in 25 mM HEPES buffer, pH 7.4, 10% Glycerol, 100 μm ZnCl_2_, 2 mM 2-mercaptoethanol. Fractions with pure His_6_-SUMO- Copia^Gag^ were pooled and dialyzed against buffer 25 mM HEPES, pH 7.4, 600 mM NaCl, 10% Glycerol, 100 μm ZnCl_2_, 2 mM 2-mercaptoethanol at 4°C overnight. The His_6_-SUMO tag was then cleaved off by Ulp1 protease and removed by Ni-affinity chromatography. The purified Copia^Gag^ protein (RNA free, A260/280 = 0.6) was dialyzed against storage buffer 25 mM HEPES, pH 7.4, 600 mM NaCl, 20% Glycerol, 100 μm ZnCl_2_, 2 mM 2-mercaptoethanol, flash frozen in liquid nitrogen and stored at −80°C.

To monitor capsid assembly by size exclusion, Ulp1 protease was added to the purified His_6_-SUMO- Copia^Gag^/His_6_-SUMO- Copia^GagΔNC^ protein after Ni-affinity purification to cleave off the His_6_-SUMO. After overnight Ulp1 digestion, the sample was loaded onto the gel filtration column (Superose 6 Increase 10/300 GL, Cytiva # 29091596) and eluted in buffer 25 mM HEPES, pH 7.4, 150 mM NaCl, 10% Glycerol, 100 μm ZnCl_2_, 500 mM Imidazole, 2 mM 2-mercaptoethanol. Assembled capsids elute at fraction 7 (retention volume = 7 ml), while the unassembled Copia^GagΔNC^ protein at fraction 24 to 26 (retention volume = from 17 to 18 ml). Ulp1 protease was added after mixing His_6_-SUMO- Copia^Gag^ and His_6_-SUMO- Copia^GagΔNC^. After overnight Ulp1 digestion, the protein mixture was loaded onto the gel filtration column (as described above), and capsids were eluted off the column at fraction 7.

### Negative-stain transmission electron microscopy

Copia^Gag^ capsid protein was concentrated using a 100 kDa MWCO Amicon Ultra-15 Centrifugal Filter Unit (EMD Millipore) to a final concentration of 0.8 mg/ml. Protein concentration was determined by Bradford assay. Copia^Gag^ capsid protein was filtered by 0.22 μm pore size PVDF membrane filter prior to deposition on the grid for EM imaging. Copper grids coated with carbon film (Electron Microscopy Sciences, CF200-Cu-50) were glow discharged on a PELCO easiGlow (Ted Pella) at 25 mA for 35 s (negative polarity) before use; 7 μl of sample was applied to the grid and incubated for 1 min. Excess sample was blotted on filter paper, then the grid was rinsed with 15 μl water (filtered by 0.22 μm PVDF membrane) 3 times followed by staining with 1% uranyl acetate (pH 4.5) for 1 min and then blotted dry. Samples were imaged with a FEI Tecnai Spirit 12 transmission electron microscope at 120 kV equipped with a Gatan 4K camera. Copia^Gag^ (RNA free, A260/A280 = 0.6) capsid protein was concentrated using a 100 kDa MWCO Amicon Ultra-15 Centrifugal Filter Unit (EMD Millipore) to a final concentration of 1.4 mg/ml. Protein was diluted 4-fold with 25 mM HEPES buffer, pH 7.4, 10% Glycerol, 100 μm ZnCl_2_, 2 mM 2-mercaptoethanol and filtered by 0.22 μm PVDF membrane. Capsid formation was assessed and imaged using negative-staining EM as described above.

### Quantification of copia capsids

Capsids were quantified using Tunable resistive pulse sensing (TRPS) for size and concentration using the Exoid system (Izon Sciences) following manufacturer recommendations. Briefly, the nanopore was set up, characterized, and wetted. Nanopore coating for biological samples was done with the Izon coating solution (Izon Sciences). Measurement of the samples was taken alongside calibration readings and the data analyzed for multi-pressure analysis using the Izon data suite (Izon Sciences).

### Cryo-EM specimen preparation

Copia^Gag^ capsid protein was dialyzed against Cryo-EM buffer (25 mM HEPES, pH 7.4, 150 mM NaCl, 100 μm ZnCl_2_, 2 mM 2-mercaptoethanol) overnight at 4°C. The protein sample was concentrated using 100 kDa MWCO Amicon Ultra-15 Centrifugal Filter Unit (EMD Millipore) to a final concentration of ~0.8 mg/ml and filtered by 0.22 μm pore size PVDF membrane prior to deposition on the grid for EM imaging. Grids were washed by ethyl acetate and glow discharged on a PELCO easiGlow (Ted Pella) at 25 mA for 35 s (negative polarity) before 3.5 μl of Copia^Gag^ capsid protein sample was applied to a 300-mesh copper grid with continuous 2 nm carbon film (Electron Microscopy Sciences, QUANTIFOIL C2-C15nCu30-50) at 10°C with 90% humidity in a Vitrobot Mark IV (FEI). Sample was blotted from both sides for 8 s with blot force 0, then immediately vitrified by plunging into liquid ethane.

### Data collection

Micrographs were collected on a 200 kV Talos-Arctica electron microscope (FEI) equipped with a K3 Summit direct electron detector (Gatan). Images were collected at a magnification of 45,000× in counting mode with an unbinned pixel size of 0.87 Å and a total dose of 39.98 e/Å^2^ per micrograph, with a target defocus range of −0.3 to −2.9 μm. In total, 10,280 micrographs were collected.

### Data processing

Copia^Gag^ capsid image processing and reconstruction are summarized in S2 and S3 Figs. Data set parameters are presented in S2B Fig. All the reconstruction steps were done within the CryoSPARC package [35]. Acquired micrograph frames were imported and motion corrected using patch-based motion correction followed by Contrast Transfer Function (CTF) estimation using patch CTF in CryoSPARC. Particles were picked using the template picker function. The template was generated by a training data set of manually picked ~80 particles.

A total of 102,206 particles were automatically picked using template picking and extracted into 896 × 896 pixel boxes (0.87 Å/pixel). Particles were binned to 224 × 224 pixel boxes (3.48 Å/pixel) during the extraction. Three rounds of 2D classification were performed to remove bad particles, resulting in a stack of 8,334 particles. We manually curated the 8,334, resulting in a stack of 6,290 particles. After one more round of 2D classification, 5,923 (94%) particles displayed icosahedral capsid structure and were selected as the final particle stack for further 3D reconstruction. The initial model was generated by ab initio reconstruction with no symmetry enforced. The ab initio model was further used in homogeneous refinement of the whole capsid with enforced Icosahedral symmetry (I) and Ewald Sphere curvature correction. The effective resolutions of the cryo-EM density maps were estimated by Fourier shell correlation (FSC = 0.143) between the 2 half maps (S2 and S3 Figs).

To further improve the resolution, we performed symmetry expansion as implemented in CryoSPARC. Local refinement at the 5-fold pentamer, the 3-fold hexamer, and the non-symmetry related hexamer within the asymmetric unit was performed using symmetry expanded particles with the rest of the density subtracted. Local refinement was performed with no symmetry applied, and we determined the structures with resolutions of 3.29 Å for the 5-fold pentamer, 3.41 Å for the 3-fold hexamer and 3.47 Å for the non-symmetric hexamer (S3 Fig).

### Model building

The structures of Copia^Gag^ capsid (CA domain: residues 1 through 186) were determined by model building into the locally refined maps of the 5-fold pentamer, 3-fold hexamer, and the non-symmetric hexamer. No structures were determined for the inner layer that is likely corresponding to the NC domain (residue 186–270) and the associated nucleic acid, due to its disordered nature.

The initial model of Copia^Gag_NTD^ (residue1-90) and Copia^Gag_CTD^ (residue 91–186) were predicted using AlphaFold2 [36] which were separately fit as rigid bodies into the map density. The initial model was built using Coot [37] and refined in Phenix [38] using real-space refinement with rotamer, Ramachandran, and secondary structure restraints. For the 5-fold and 3-fold capsomers, symmetry constraints were applied during the refinement. The refinement and validation statistics were gained from the refinement report on Phenix. ChimeraX was used for molecular visualization and analysis [39].

### Isolation and quantification of EVs from S2 cells

EVs were isolated from S2 cells cultured in serum-free medium at 22°C. EVs-containing media was first centrifuged at 500×g for 5 min to pellet the cells, the supernatant was then spun at 2,000×g for 10 min at 4°C to eliminate cell debris, and to retain small EVs the samples were further centrifuged at 10,000×g for 30 min at 4°C. The supernatants were filtered with a 0.22 μm PES filtration unit (EMD Millipore), the samples were concentrated with a Centricon centrifugal filter (EMD Millipore) and the EV-rich filtride recovered. The samples were then purified by size exclusion chromatography using qEV isolation columns (Izon Sciences) on an automatic fraction collector (Izon Science), the fractions were then pooled based on protein concentration and using Amicon centrifugal filters (EMD Millipore) concentrated the samples. Samples for quantification were diluted in PBS and as previously described analyzed for size and concentration using the Exoid TRPS system (Izon Science). Unused samples were stored at –80°C.

### Transmission electron microscopy of S2 cells EVs

EVs were fixed in 2% paraformaldehyde overnight at 4°C and 5 μl was spotted onto formvar coated grids for 5 min. The grids were wicked using Whatman filter paper, rinsed with PBS, and post-fixed with 1% glutaraldehyde for 5 min. The protocol was completed as described above for capsids.

### S2 cells derived EVs immuno-electron microscopy

Samples were prepared as previously described [5]. In brief, the EV preparations were fixed overnight at 4°C in 2% PFA (EMS) and 5 μl of sample applied to formvar coated gold grids (EMS) and incubated for 5 min. Grids were wicked on Whatman #50 filter paper (GE Healthcare) after which they were washed in 100 mM Tris followed by additional washes in 100 mM Tris + 50 mM Glycine. Grids were blocked for 10 min in blocking buffer (EMS) and either incubated in Tris (control) or lysed with 0.05% saponin in Tris. After washing in Tris, they were incubated in primary antibody for 1 h, washed in Tris, and then incubated with anti-rabbit and/or anti-mouse conjugated to 10 nm, 15 nm, or 18 nm gold secondary antibodies (EMS). Grids were washed, post fixed with 1% glutaraldehyde, washed in water, and finally negative stained with 1% uranyl acetate for 30 s. Grids were imaged on an FEI Tecnai 12 Spirit equipped with a Gatan 4K camera.

### RNA sequencing

RNA was isolated as outlined in the immunoprecipitation section. Samples were kept in RLT buffer (QIAGEN) with 20 μl 2 M dithiothreitol (DTT) per 1 ml RLT buffer pre-added and homogenized, RNA was extracted with RNeasy micro-Kit (QIAGEN) according to the extraction protocol without any modifications. RNA concentration and integrity were determined by Qubit 4 Fluorometer (Thermo Fisher Scientific) and 2100 Bioanalyzer (Agilent), respectively. RNA libraries were made with NEBNext Ultra II Directional RNA Library Prep Kit for Illumina kit without any modifications. The total RNA for each library was about 500 ng, 9 PCR cycles were used for library amplification. The concentration and quality of libraries were assessed with Qubit 4 Fluorometer (Thermo Fisher Scientific) and 2100 Bioanalyzer (Agilent), respectively. Each library was normalized to 20 nM and pooled first, then normalized to the final concentration of 5 nM and loaded onto NextSeq500/550 High Output Kit v2.5 (150 Cycles) chip (Illumina) on the sequencer NextSeq550 (Illumina). Sequencing data was uploaded to SRA PRJNA1120037.

Reads were sorted by barcode for each library and adapter sequences removed by Trimmomatic [48]. Reads were mapped to Drosophila genome by STAR [49]. The transcript expressions were counted by TEtranscripts [50], and the differential expression genes were explored by DeSeq2 [51].

### Bioinformatic analysis

All code and data needed to reproduce these analyses can be found here:

https://github.com/alfredsimkin/copia_regulates_plasticity.

### TE sequences in mRNAs (i.e., off-targeting of RNAi constructs)

To search for TE sequences within mature or immature mRNAs, we obtained sequences of the dm6 genome (see above), 34,463 transcripts corresponding to all mature mRNA isoforms of all annotated RefSeq genes, and the corresponding immature mRNAs from the same transcripts from the UCSC genome browser. We used the “table browser” tool of UCSC genome browser to obtain these transcripts and selected the “RefSeq All” table of the “NCBI RefSeq” track of the “Genes and Gene Predictions” group. We collapsed individual immature mRNA transcripts into nonredundant genomic regions. We searched for *Copia*^*pol*^-shRNA and *Copia*^*gag*^-siRNA regions in both the dm6 genome and in mature mRNA transcripts. We also parsed out genomic coordinates from the names of our 38 full-length Copia seeds (see above). We define an RNAi region of the genome as “inside a Copia seed” if its genomic coordinates are fully contained within the genomic coordinates of a full-length Copia seed, and we define an RNAi region of the genome as “inside an immature Refseq transcript” if its coordinates are fully contained within an immature Refseq transcript. We define an RNAi region as “inside a mature mRNA” if blat finds any matching nucleotides of the RNAi region within a transcript of the Refseq mature mRNA database. No immature or mature mRNAs contains more than one *Copia* si/shRNA target sequence and a chimeric gene sequence, as the phenotypes which we see with more than one si/shRNA sequence cannot be explained through an indirect knockdown of a sequence chimeric with *Copia*.

### Quantification and statistical analysis

Statistical analyses for single comparisons were performed using a Student’s *t* test while multiple comparisons with experimental groups utilized a one-way analysis of variance (ANOVA) with the appropriate post hoc test. *, *p* < 0.05; **, *p* < 0.001; ***, *p* < 0.0001. Raw data files were processed with Excel (Microsoft) and data analysis for statistical significance utilized GraphPad Prism version 9.5.0 (GraphPad Software).

### Inclusion and diversity

One or more of the authors of this paper self-identifies as an underrepresented ethnic minority in science. We support inclusive, diverse, and equitable conduct of research.

## Supporting information

S1 Fig Copia protein and mRNA are enriched in *Drosophila* larval CNS. (A) Schematic of Copia^gag^. The orange bar represents the region of Copia^gag^ used to generate a Copia^full^ antibody. (B) Peptide competition assay. On the left, lysates (labelled below) are probed with α-Copia^gag^. On the right, the blot is incubated with the Copia^gag^ antigen, α-Copia^gag^. α- GAPDH staining is used as a negative control. (C) Close up of a single bouton labelled with the presynaptic marker HRP, postsynaptic marker DLG, and α-Copia^gag^. A cartoon derived from this micrograph accentuates the defined presynaptic (red-HRP) and postsynaptic (blue-DLG) compartments. (D–H) The knockdown of Copia in the muscle utilizing the C57 driver results in reduction of Copia^gag^ signal in the postsynaptic region as quantified in G and H. (I) Bacterially expressed Copia^gag^ self-assembles into capsid-like structures observable using negative stain EM. Scale bar = 1,000 nm. Inset: close up of an individual capsid, scale bar = 50 nm. (J) Graph of particle counts (Izon Exoid) from bacterially expressed Copia^gag^. The data underlying the graphs shown in the figure can be found in S1 Data, raw gel images can be found in S1 Raw Images. *N* = number of NMJs in G and H (by genotype from left to right) 6, 7 and 7. ns *p* ≥ 0.05, * *p* < 0.05, ** *p* < 0.01, *** *p* < 0.001, and **** *p* < 0.0001.
(TIFF)

S2 Fig Copia associates with its own transcript and that of dArc1 while in mutually exclusive structures. (A–D) RNA immunoprecipitation using antibodies as labelled on the X-axis, probing for Copia^full^ mRNA (A) and Copia^gag^ mRNA (C) from larval CNS, while probing for the same targets in D but with RNA-IPed from BWM. (E, F) Rpl32 control for pre-immune of α- Copia^full^ and α- Copia^gag^ antibodies. (G) EVs isolated from S2 cells were immuno-stained with α-Copia^gag^ (10 nm) and α-Syntaxin 1A (15 nm) and imaged with EM scale bar = 50 nm. (H) EVs isolated from S2 cells were treated with detergent to dissociate EV membranes, leaving capsids accessible to antibodies. Yellow arrows are unlabeled structures, blue arrow points to an electron dense structure presumably a capsid labelled with α-Copia^gag^ (18 nm) and red arrow points to a α-dArc1 (10 nm) labeled capsid, scale bar = 200 nm. (I) Secondary antibody alone control shows little or no gold-particle labels, scale bar = 200 nm. (J) Close-up of an electron density labelled with Copia antibody, scale bar = 50 nm. (K–N) Fold enrichment of targets following immunoprecipitation and RNA-seq. Antibodies indicated on the x-axis and RNA of interest on the y-axis. (O, P) dPCR of immunoprecipitation using α-Copia^full^ or α-Copia^gag^ with dArc1 being the RNA probed. The data underlying the graphs shown in the figure can be found in S1 Data.
(TIFF)

S3 Fig Assembly requirements for Copia^gag^. (A) Copia^gag-PR^ auto-processes to cleave off the Protease (PR) region. Subsequent removal of the His_6_-SUMO tag triggers assembly into the capsid form. (B) Auto-processing in cellulo. Uninduced *E*. *coli* cells (UI) show no expression of Copia^gag+PR^. After 4 h of expression at 18°C, substantial full-length Copia^gag+PR^ is observed, but after overnight expression nearly all Copia^gag+PR^ is autoprocessed into Copia^gag^ and Copia^PR^. (C) Removal of RNA through ion exchange chromatography results in a Copiag^ag^ that does not assemble into capsids. (D) Monitoring capsid assembly by Size Exclusion Chromatography. The capsid form of Copia^gag^ elutes in fraction 7, while unassembled protein elutes in later fractions. (E) A construct that lacks the RNA-binding Nucleocapsid domain (Copia^gagΔNC^) does not assemble into capsids. (F) A mixture of Copia^gag^ and Copia^gagΔNC^ results in both proteins assembling into capsids. This result illustrates that Copia^gagΔNC^ is assembly-competent, but lacks the ability to trigger assembly in isolation, presumably because of the lack of bound RNA. Raw gel images can be found in S1 Raw Images.
(TIFF)

S4 Fig Cryo-EM structure determination of Copia^Gag^ capsid. All data processing was performed using cryoSPARC. (A) Workflow for cryo-EM structure determination. Particles were first manually picked, which then were used for the template picker function. Particles were extracted and underwent 3 rounds of 2D classification, followed by manual curation and another round of 2D classification. (B) Icosahedral symmetric structure determination. To obtain a structure of the complete capsid, we then performed 3D reconstruction with icosahedral symmetry enforced, and the outer layer of density masked. Local resolution of the reconstructions and a representative section of each density map are shown. The overall resolution of each map was determined by the FSC of each half-map using Gold-standard cutoff of 0.143. (C) Table of Cryo-EM data collection, processing, and model statistics. (D) Structure determination of individual capsomers. To obtain high-resolution, symmetry expansion was used to isolate individual capsomers. After signal subtraction and masking the outer layer, the reconstruction of each capsomer was refined locally. Fourier shell correlation (FSC) was used to estimate the overall resolution of each reconstruction (FSC = 0.143 cutoff) and a representative section of each density map is shown. The data underlying the structures shown in the figure can be found in S1 Data.
(TIFF)

S5 Fig Comparison of Copia capsid with other retroelement-derived capsids. (A) Comparison of Copia and dArc1 capsid electrostatics. Individual subunits of Copia (left) and dArc1 (PDB ID 6TAP) are shown with the primary interaction surface colored by electrostatic potential. The electrostatic potential of Copia and dArc1 capsid proteins are vastly different, suggesting that these proteins would not interact with each other. (B) Comparison of Copia with Gypsy retrotransposon, HIV, and dArc1 capsids. The top panel is a schematic of the overall morphology of each capsid. Both Copia and Gypsy adopt T = 9 icosahedral geometry, while HIV capsids are cone-shaped and dArc1 capsids form T = 4 icosahedral symmetry studded with spike protrusions. The middle panel is a schematic representing individual hexameric capsomers from each capsid. The bottom panel shows the protein model built into the maps. (Note that dArc1 spikes were not modeled in the dArc1 structure and thus are not shown here.) HIV stabilizes subunit–subunit interactions through an extensive interaction between the NTD of one subunit with the CTD of the previous subunit. dArc1 capsomers are stabilized by the extensive spike protrusions that help multimerize the capsomer. Copia and Gypsy lack these stabilization elements but have evolved a smaller interaction surface between adjacent CTDs that is not found in HIV or dArc1.
(TIFF)

S6 Fig Double mutations of *Copia* and *dArc1* show the Copia phenotype is predominant at the *Drosophila* larval NMJ. (A–C) *dArc1* null (trans-heterozygous) flies (B1) show a substantial reduction in bouton formation compared to wild-type controls (A). (C) There is a striking increase in bouton numbers (C1), but not hyperbudding (C2) in flies that are overexpressing *dArc1* presynaptically. (D, E) Flies presynaptically expressing either *Copia*^pol^-shRNA (D) or *Copia*^gag^-siRNA (E) in an *dArc1* null background have increased number of boutons and hyberbudding. (F, G) Comparison of bouton number and hyperbudding in wild type, *dArc1* null, *dArc1* OE, and *dArc1* null expressing knockdown constructs against either *Copia*^pol^ or *Copia*^gag^ in neurons. (H) A summation of the genetic interactions between dArc1 and Copia, whereby dArc1 and Copia combat each other to control plasticity, altogether these genetic interactions suggest the NMJ is programmed to be at a high state of plasticity (++plasticity++). (I–K) A model for the interaction between Copia and dArc1. The NMJ is in a state of high potential for plasticity and Copia represses plasticity and is predominant to dArc1 and as such removing both from the NMJ results in increased plasticity. Copia and dArc1 capsids compete for dArc1 mRNA. Illustrated in panel I, Copia capsids sequester *dArc1* mRNA, leading to a reduction in synaptic plasticity. In contrast, in J, a reduction of Copia binding to *dArc1* mRNA leads to an increase in dArc1 capsids and increased plasticity. In panel K, there is a balance of Copia and dArc1. The data underlying the graphs shown in the figure can be found in S1 Data. DLG = α-Discs Large (postsynaptic marker), HRP = α-horseradish peroxidase (presynaptic marker). N = (by genotype from top to bottom; number of animals/NMJs quantified) 9/17, 7/12, 12/22, 4/8, 6/11 in (F) and (G). Full genotypes in Materials and methods. ns *p* ≥ 0.05, * *p* < 0.05, ** *p* < 0.01, *** *p* < 0.001, and **** *p* < 0.0001.
(TIFF)

S1 Data Data underlying Figs 1F–1I, 2G, 3F–3K, 5D, 5E, S1G, S1H, S1J, S2A–S2F, S2K–S2P, S4B, S4D, S6F, S6G, and sequences for constructs and probes mentioned in Methods.
(XLSX)

S1 Raw Images PDF file with all uncropped gels and blots corresponding to data in Figs 1B, S1B, S3B and S3D–S3F.
(PDF)

## Abbreviations

BWM: body wall muscle
CNS: central nervous system
CTD: C-terminal domain
CTF: Contrast Transfer Function
EV: extracellular vesicle
HIV: human immunodeficiency virus
NMJ: neuromuscular junction
NPC: nuclear pore complex
NTD: N-terminal domain
PBS: phosphate-buffered saline
RIP: RNA immunoprecipitation
TE: transposable element
TRPS: tunable resistive pulse sensing
