# Supplementary figures and images for: Capsid transfer of the retrotransposon Copia controls structural synaptic plasticity in Drosophila
Source: PLoS Biol. 2025 Feb 18;23(2):e3002983. doi: 10.1371/journal.pbio.3002983 (PMC11835246; doi:10.1371/journal.pbio.3002983)

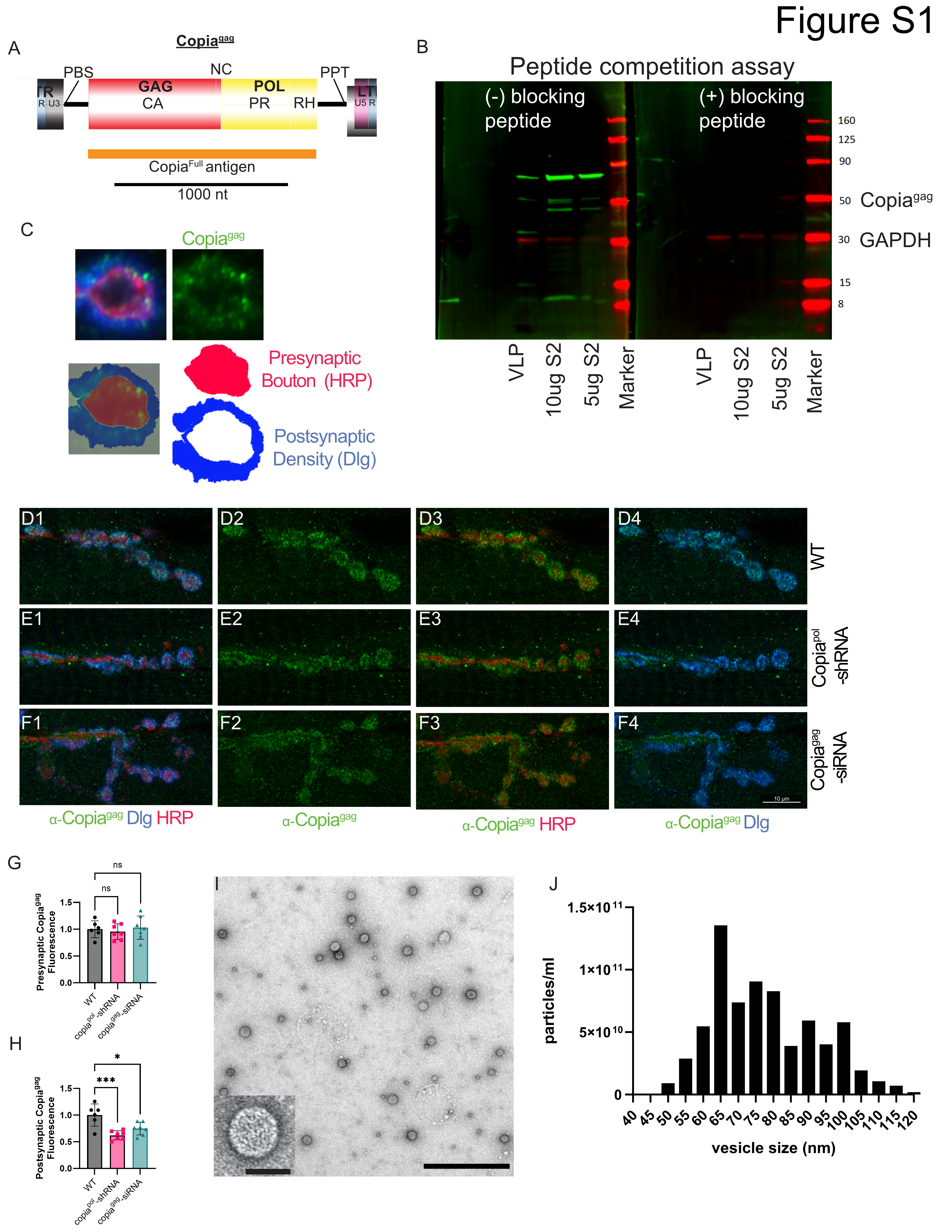

Supplement: S1 Fig — (A) Schematic of Copiagag. The orange bar represents the region of Copiagag used to generate a Copiafull antibody. (B) Peptide competition assay. On the left, lysates (labelled below) are probed with α-Copiagag. On the right, the blot is incubated with the Copiagag antigen, α-Copiagag. α- GAPDH staining is used as a negative control. (C) Close up of a single bouton labelled with the presynaptic marker HRP, postsynaptic marker DLG, and α-Copiagag. A cartoon derived from this micrograph accentuates the defined presynaptic (red-HRP) and postsynaptic (blue-DLG) compartments. (D–H) The knockdown of Copia in the muscle utilizing the C57 driver results in reduction of Copiagag signal in the postsynaptic region as quantified in G and H. (I) Bacterially expressed Copiagag self-assembles into capsid-like structures observable using negative stain EM. Scale bar = 1,000 nm. Inset: close up of an individual capsid, scale bar = 50 nm. (J) Graph of particle counts (Izon Exoid) from bacterially expressed Copiagag. The data underlying the graphs shown in the figure can be found in S1 Data, raw gel images can be found in S1 Raw Images. N = number of NMJs in G and H (by genotype from left to right) 6, 7 and 7. ns p ≥ 0.05, * p < 0.05, ** p < 0.01, *** p < 0.001, and **** p < 0.0001. (TIFF) [file pbio.3002983.s001.tiff]

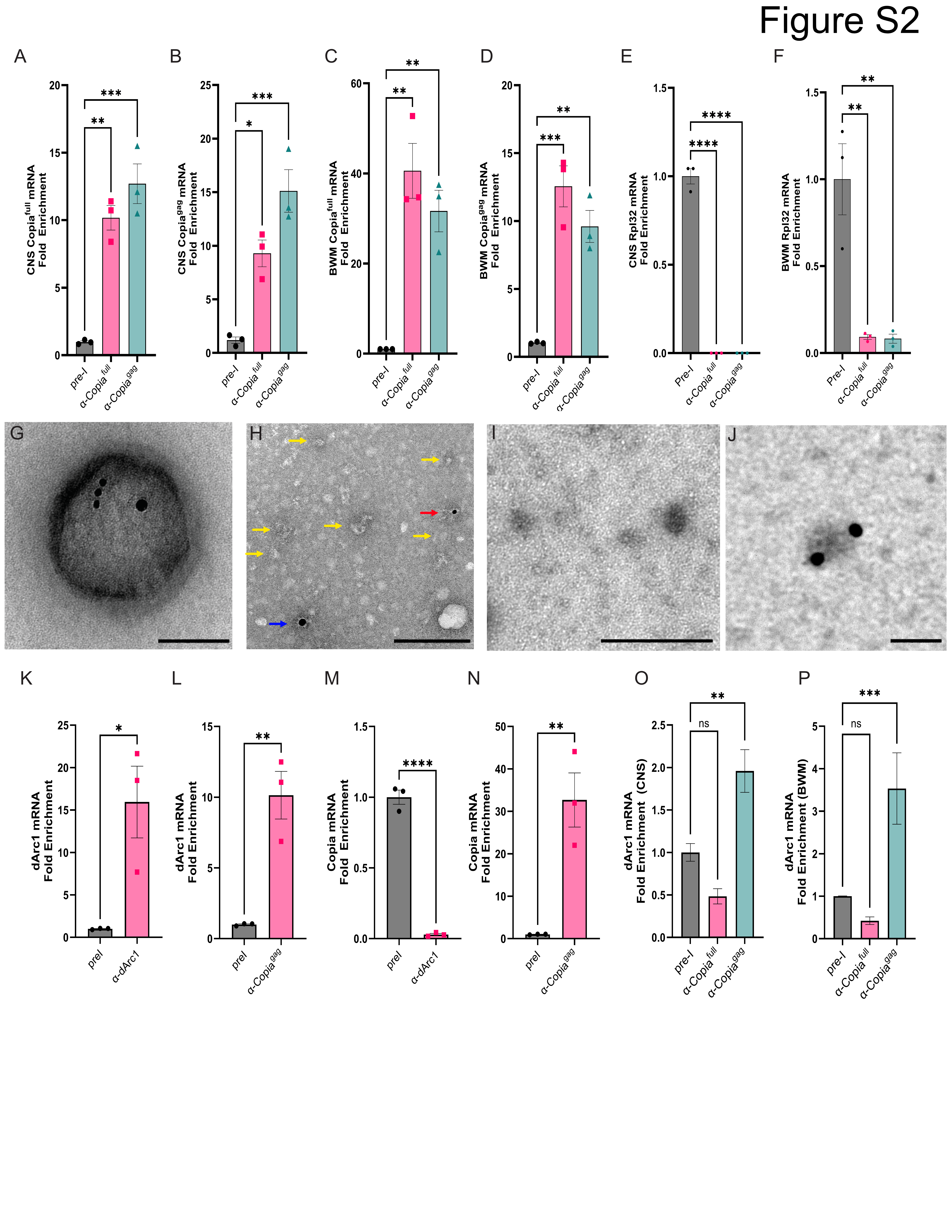

Supplement: S2 Fig — (A–D) RNA immunoprecipitation using antibodies as labelled on the X-axis, probing for Copiafull mRNA (A) and Copiagag mRNA (C) from larval CNS, while probing for the same targets in D but with RNA-IPed from BWM. (E, F) Rpl32 control for pre-immune of α- Copiafull and α- Copiagag antibodies. (G) EVs isolated from S2 cells were immuno-stained with α-Copiagag (10 nm) and α-Syntaxin 1A (15 nm) and imaged with EM scale bar = 50 nm. (H) EVs isolated from S2 cells were treated with detergent to dissociate EV membranes, leaving capsids accessible to antibodies. Yellow arrows are unlabeled structures, blue arrow points to an electron dense structure presumably a capsid labelled with α-Copiagag (18 nm) and red arrow points to a α-dArc1 (10 nm) labeled capsid, scale bar = 200 nm. (I) Secondary antibody alone control shows little or no gold-particle labels, scale bar = 200 nm. (J) Close-up of an electron density labelled with Copia antibody, scale bar = 50 nm. (K–N) Fold enrichment of targets following immunoprecipitation and RNA-seq. Antibodies indicated on the x-axis and RNA of interest on the y-axis. (O, P) dPCR of immunoprecipitation using α-Copiafull or α-Copiagag with dArc1 being the RNA probed. The data underlying the graphs shown in the figure can be found in S1 Data. (TIFF) [file pbio.3002983.s002.tiff]

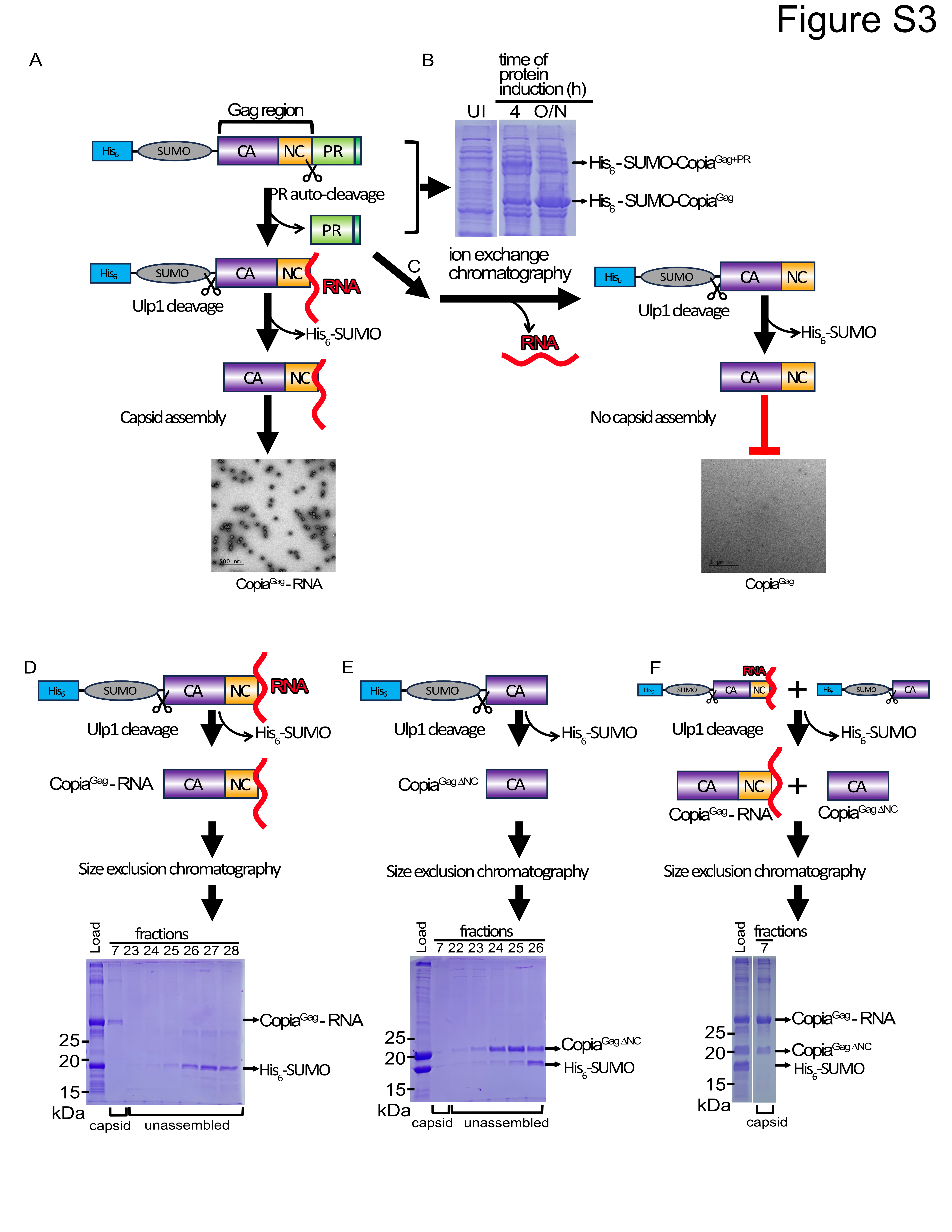

Supplement: S3 Fig — (A) Copiagag-PR auto-processes to cleave off the Protease (PR) region. Subsequent removal of the His6-SUMO tag triggers assembly into the capsid form. (B) Auto-processing in cellulo. Uninduced E. coli cells (UI) show no expression of Copiagag+PR. After 4 h of expression at 18°C, substantial full-length Copiagag+PR is observed, but after overnight expression nearly all Copiagag+PR is autoprocessed into Copiagag and CopiaPR. (C) Removal of RNA through ion exchange chromatography results in a Copiagag that does not assemble into capsids. (D) Monitoring capsid assembly by Size Exclusion Chromatography. The capsid form of Copiagag elutes in fraction 7, while unassembled protein elutes in later fractions. (E) A construct that lacks the RNA-binding Nucleocapsid domain (CopiagagΔNC) does not assemble into capsids. (F) A mixture of Copiagag and CopiagagΔNC results in both proteins assembling into capsids. This result illustrates that CopiagagΔNC is assembly-competent, but lacks the ability to trigger assembly in isolation, presumably because of the lack of bound RNA. Raw gel images can be found in S1 Raw Images. (TIFF) [file pbio.3002983.s003.tiff]

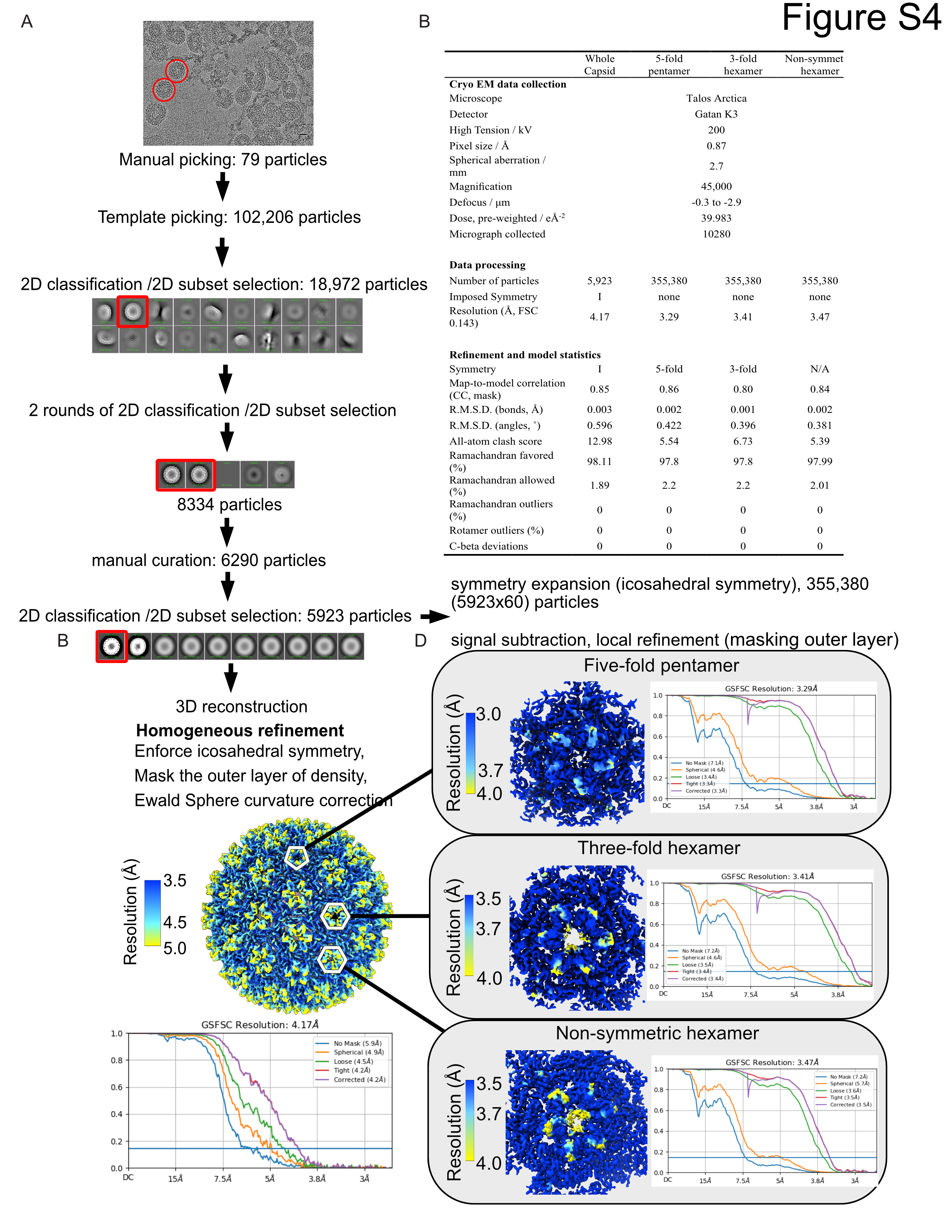

Supplement: S4 Fig — All data processing was performed using cryoSPARC. (A) Workflow for cryo-EM structure determination. Particles were first manually picked, which then were used for the template picker function. Particles were extracted and underwent 3 rounds of 2D classification, followed by manual curation and another round of 2D classification. (B) Icosahedral symmetric structure determination. To obtain a structure of the complete capsid, we then performed 3D reconstruction with icosahedral symmetry enforced, and the outer layer of density masked. Local resolution of the reconstructions and a representative section of each density map are shown. The overall resolution of each map was determined by the FSC of each half-map using Gold-standard cutoff of 0.143. (C) Table of Cryo-EM data collection, processing, and model statistics. (D) Structure determination of individual capsomers. To obtain high-resolution, symmetry expansion was used to isolate individual capsomers. After signal subtraction and masking the outer layer, the reconstruction of each capsomer was refined locally. Fourier shell correlation (FSC) was used to estimate the overall resolution of each reconstruction (FSC = 0.143 cutoff) and a representative section of each density map is shown. The data underlying the structures shown in the figure can be found in S1 Data. (TIFF) [file pbio.3002983.s004.tiff]

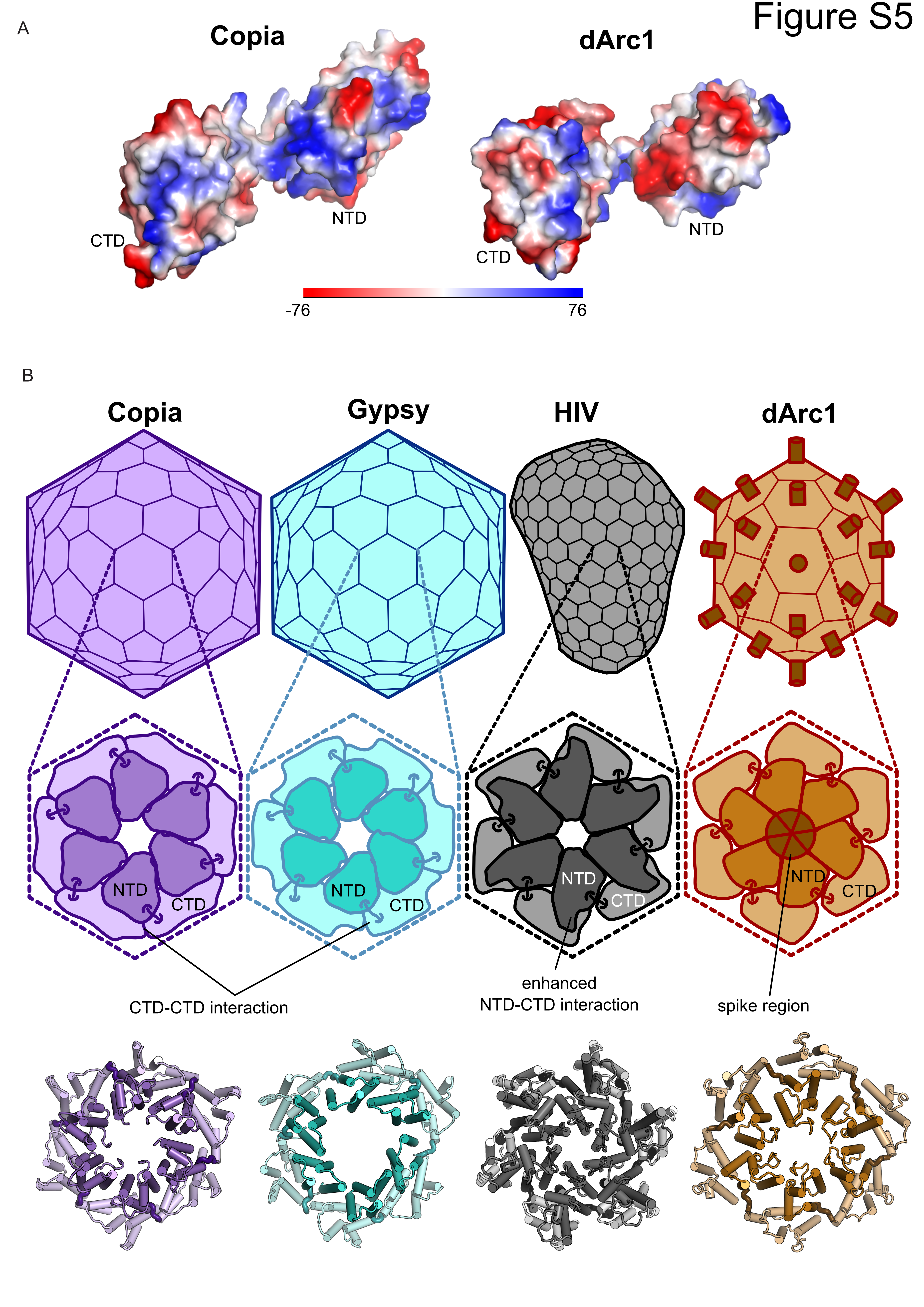

Supplement: S5 Fig — (A) Comparison of Copia and dArc1 capsid electrostatics. Individual subunits of Copia (left) and dArc1 (PDB ID 6TAP) are shown with the primary interaction surface colored by electrostatic potential. The electrostatic potential of Copia and dArc1 capsid proteins are vastly different, suggesting that these proteins would not interact with each other. (B) Comparison of Copia with Gypsy retrotransposon, HIV, and dArc1 capsids. The top panel is a schematic of the overall morphology of each capsid. Both Copia and Gypsy adopt T = 9 icosahedral geometry, while HIV capsids are cone-shaped and dArc1 capsids form T = 4 icosahedral symmetry studded with spike protrusions. The middle panel is a schematic representing individual hexameric capsomers from each capsid. The bottom panel shows the protein model built into the maps. (Note that dArc1 spikes were not modeled in the dArc1 structure and thus are not shown here.) HIV stabilizes subunit–subunit interactions through an extensive interaction between the NTD of one subunit with the CTD of the previous subunit. dArc1 capsomers are stabilized by the extensive spike protrusions that help multimerize the capsomer. Copia and Gypsy lack these stabilization elements but have evolved a smaller interaction surface between adjacent CTDs that is not found in HIV or dArc1. (TIFF) [file pbio.3002983.s005.tiff]

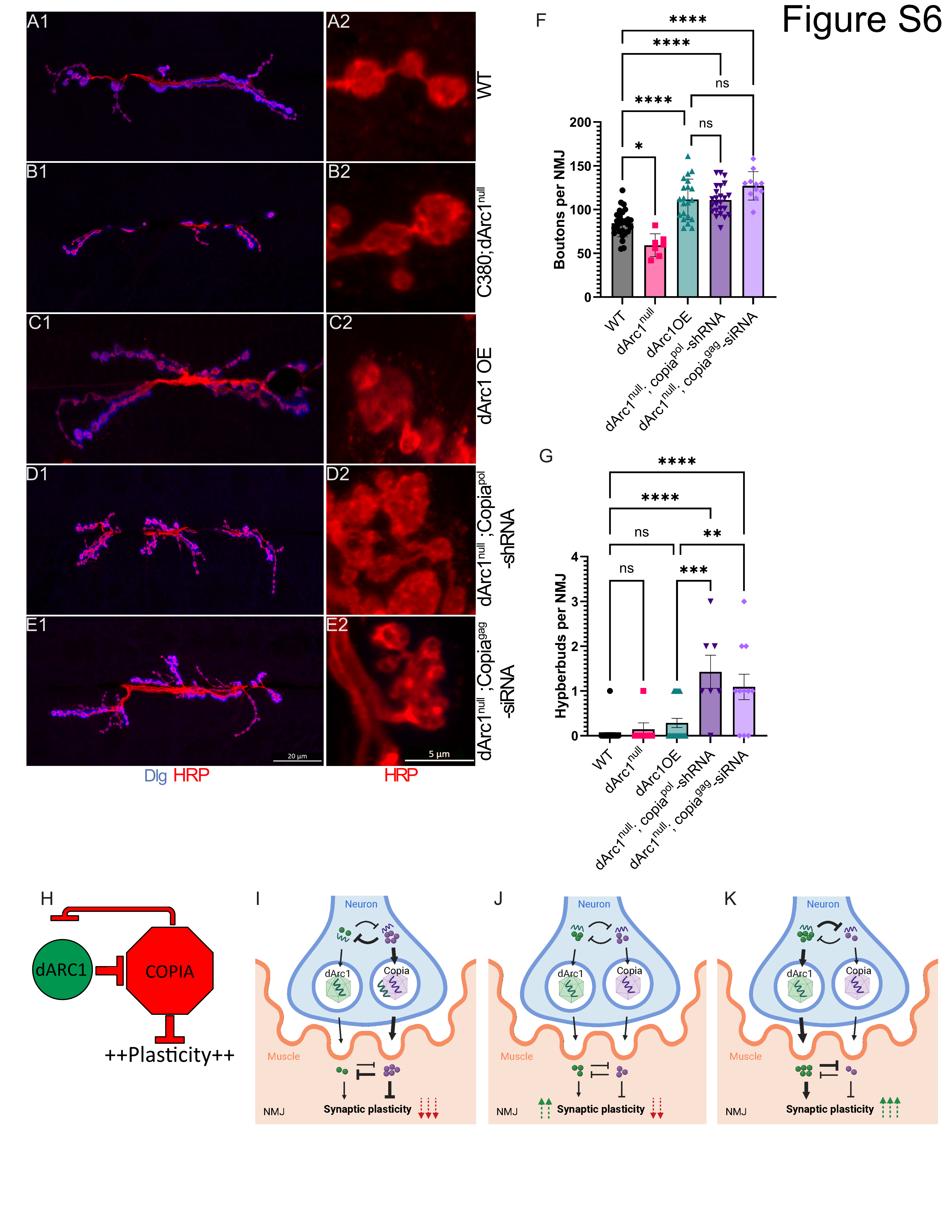

Supplement: S6 Fig — (A–C) dArc1 null (trans-heterozygous) flies (B1) show a substantial reduction in bouton formation compared to wild-type controls (A). (C) There is a striking increase in bouton numbers (C1), but not hyperbudding (C2) in flies that are overexpressing dArc1 presynaptically. (D, E) Flies presynaptically expressing either Copiapol-shRNA (D) or Copiagag-siRNA (E) in an dArc1 null background have increased number of boutons and hyberbudding. (F, G) Comparison of bouton number and hyperbudding in wild type, dArc1 null, dArc1 OE, and dArc1 null expressing knockdown constructs against either Copiapol or Copiagag in neurons. (H) A summation of the genetic interactions between dArc1 and Copia, whereby dArc1 and Copia combat each other to control plasticity, altogether these genetic interactions suggest the NMJ is programmed to be at a high state of plasticity (++plasticity++). (I–K) A model for the interaction between Copia and dArc1. The NMJ is in a state of high potential for plasticity and Copia represses plasticity and is predominant to dArc1 and as such removing both from the NMJ results in increased plasticity. Copia and dArc1 capsids compete for dArc1 mRNA. Illustrated in panel I, Copia capsids sequester dArc1 mRNA, leading to a reduction in synaptic plasticity. In contrast, in J, a reduction of Copia binding to dArc1 mRNA leads to an increase in dArc1 capsids and increased plasticity. In panel K, there is a balance of Copia and dArc1. The data underlying the graphs shown in the figure can be found in S1 Data. DLG = α-Discs Large (postsynaptic marker), HRP = α-horseradish peroxidase (presynaptic marker). N = (by genotype from top to bottom; number of animals/NMJs quantified) 9/17, 7/12, 12/22, 4/8, 6/11 in (F) and (G). Full genotypes in Materials and methods. ns p ≥ 0.05, * p < 0.05, ** p < 0.01, *** p < 0.001, and **** p < 0.0001. (TIFF) [file pbio.3002983.s006.tiff]
