# Supplementary material for: Capsid transfer of the retrotransposon Copia controls structural synaptic plasticity in Drosophila
Source: PLoS Biol. 2025 Feb 18;23(2):e3002983. doi: 10.1371/journal.pbio.3002983 (PMC11835246; doi:10.1371/journal.pbio.3002983)

Fig1B

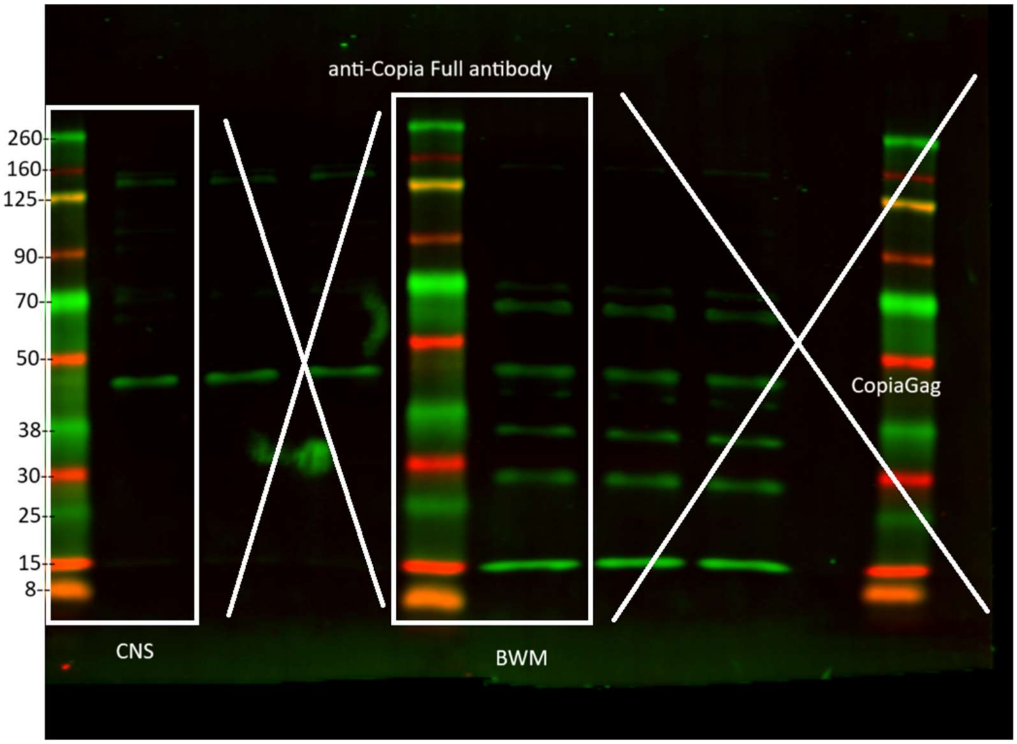
anti-Copia Full antibody
260
160
125
90
70
50
38
30
25
15
8
CopiaGag
CNS
BWM

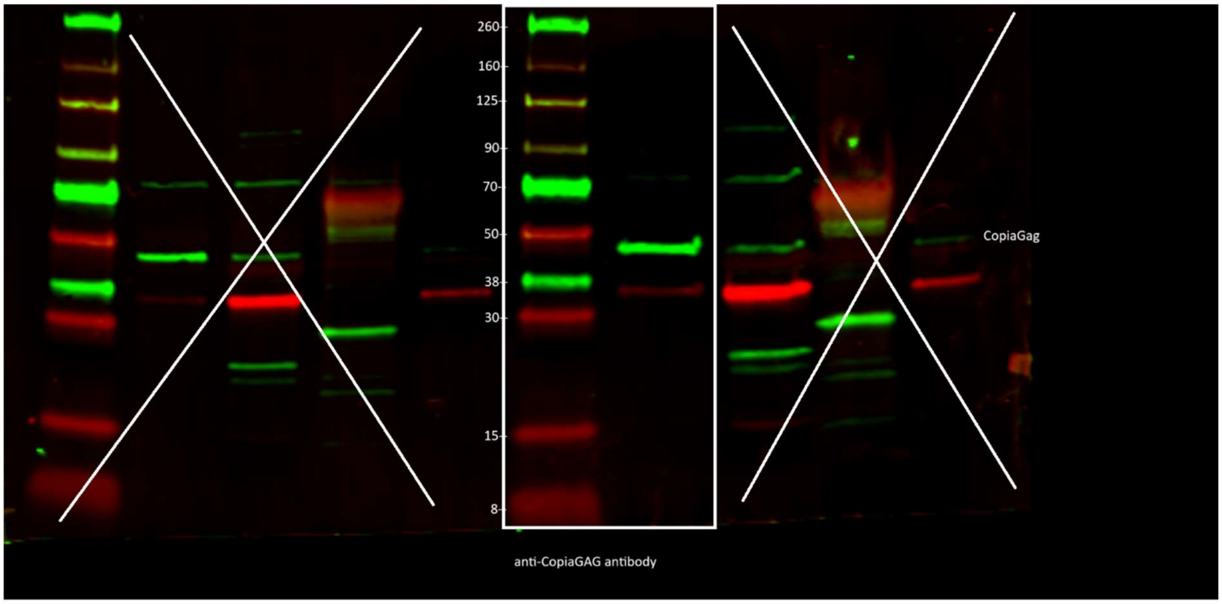
260
160
125
90
70
50
38
30
15
8
CopiaGag
anti-CopiaGAG antibody

FigS1B

(Image in supplemental figure is uncropped, original image below)

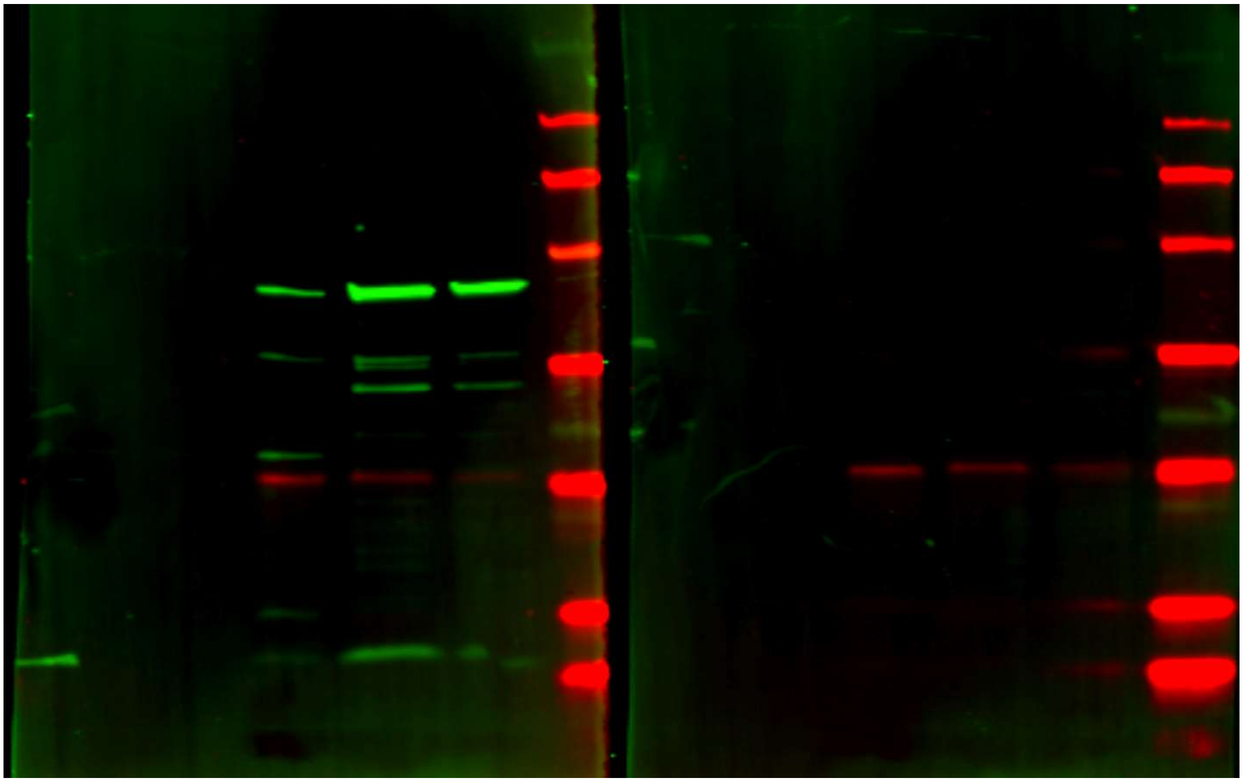

FigS3B

red frame are the gel lanes shown in the figure

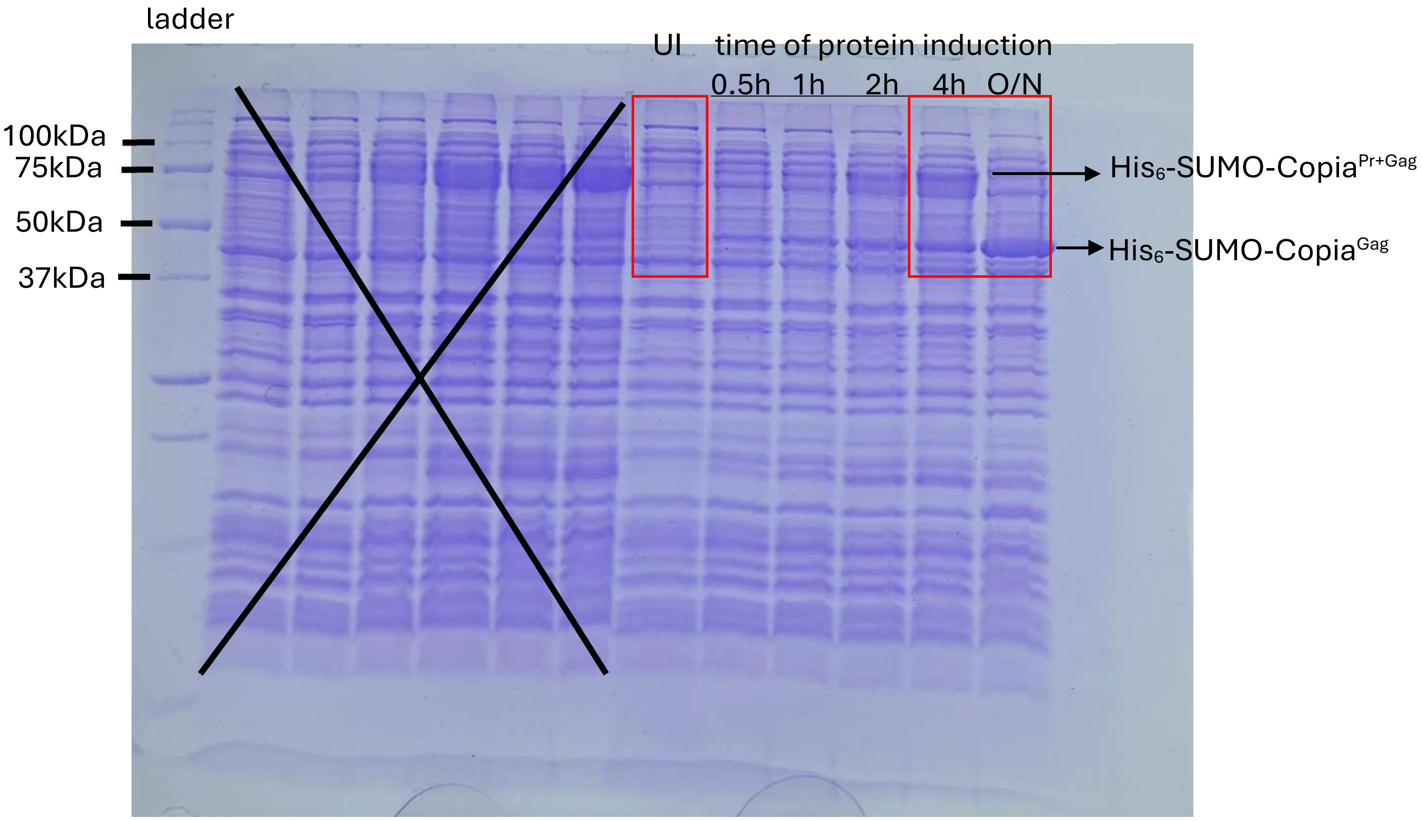

FigS3D & E
red frame are the gel lanes shown in the figure

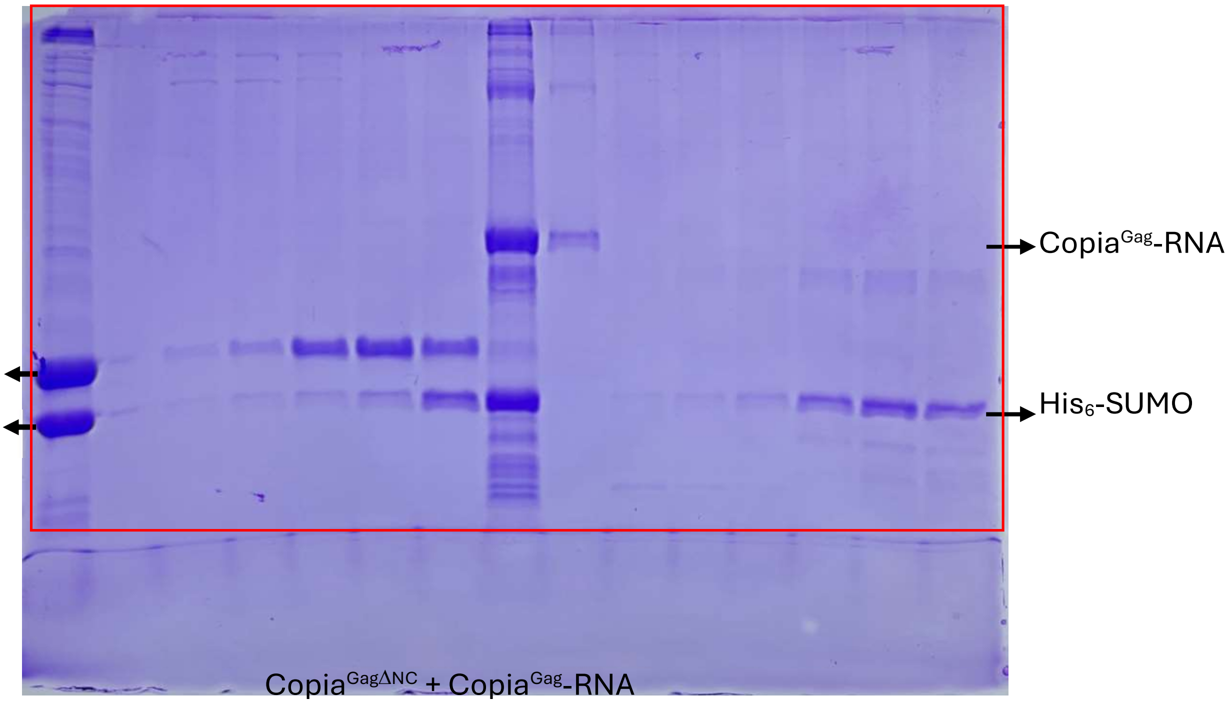

FigS3F

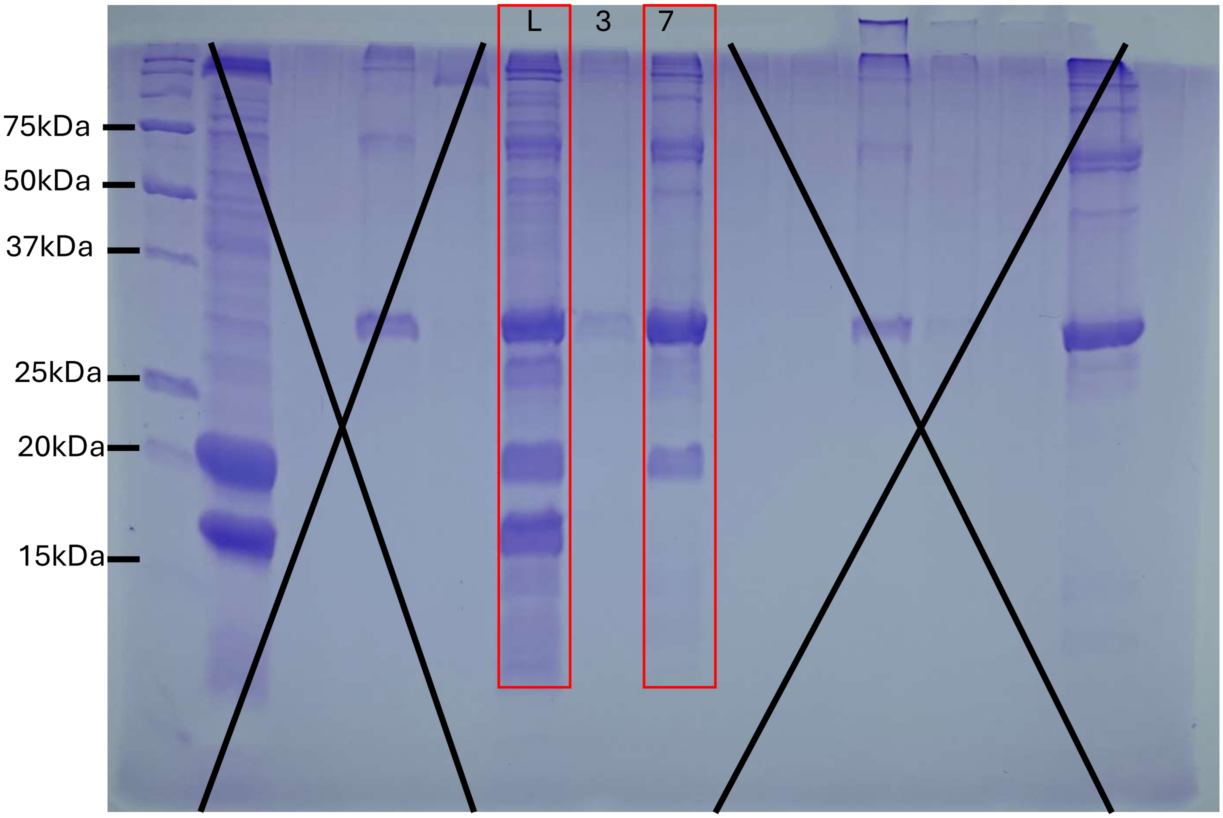

Supplement: S1 Raw Images — PDF file with all uncropped gels and blots corresponding to data in Figs 1B, S1B, S3B and S3D–S3F. (PDF) [file pbio.3002983.s008.pdf]
